# Online and traditional mindfulness-based interventions for stress in university students: a systematic review and meta-analysis versus control conditions

**DOI:** 10.3389/fpsyg.2026.1755245

**Published:** 2026-03-27

**Authors:** Yangzhen Wang, Weiguo Qu, Hongcheng Liu, Ze Peng, Yurong Hu

**Affiliations:** 1 Xiangtan Institute of Technology, Xiangtan, China; 2 Department of Psychology, School of Educational Science, Hunan Normal University, Changsha, China; 3 Changsha Institute of Technology, Changsha, China

**Keywords:** meta-analysis, online mindfulness, stress, traditional mindfulness, university student

## Abstract

**Objective:**

Empirical evidence verifies the efficacy of mindfulness interventions in addressing heterogeneous stress forms across clinical and non-clinical populations. However, online mindfulness is increasingly emerging in diverse modalities across online platforms, propelled by ongoing technological advancements. This study aims to conduct a systematic analysis of the intervention effects of online versus traditional mindfulness in alleviating stress among university students. It is important to note that this review compares online and traditional formats indirectly, by synthesizing trials that separately compare each format against a control condition, as head-to-head RCTs directly comparing both formats were not eligible for inclusion.

**Methods:**

Four databases were selected as data sources: Web of Science, Embase, Medline and PsycINFO. A total of 4,387 articles were included based on the PICOS framework established *a priori*, and 16 articles were ultimately incorporated into the meta-analysis. During data analysis, article quality was appraised utilizing the Cochrane Risk of Bias Tool, with data extraction performed in Microsoft Excel and statistical analyses conducted in Stata.

**Results:**

Analysis of the included studies indicated that online mindfulness interventions (SMD = −0.56; 95% CI: −0.76 to −0.36) effectively reduced stress in university students, with traditional mindfulness interventions (SMD = −0.64; 95% CI: −0.83 to −0.46) demonstrating comparable results. For improving mindfulness among university students, both online mindfulness (SMD = 0.52; 95% CI: 0.28 to 0.76) and traditional mindfulness (SMD = 0.42; 95% CI: 0.24 to 0.59) yield positive outcomes.

**Conclusion:**

The results demonstrated that both online and traditional mindfulness effectively reduce university students’ stress and enhance their mindfulness levels. And the effects of these two intervention methods are comparable. Therefore, online mindfulness may serve as a viable and similarly effective alternative to traditional mindfulness for stress reduction among college students.

**Systematic review registration:**

https://www.crd.york.ac.uk/PROSPERO/view/CRD420251025123, CRD420251025123.

## 1 Introduction

### 1.1 Stress and mental health in university students

With the rapid development of society, stress has become a prevalent issue experienced daily (O’Connor et al., 2021). It is closely related to mental health issues and has drawn the attention of the World Health Organization (2022). According to the data on emotional states released by Gallup in 2024, people’s negative emotions such as stress and sadness have reached the highest level since the organization began its investigations (Gallup, 2024).

The university years represent a crucial transitional period for entering society. Students encounter multiple challenges such as changes in living environment, shifts in social roles, and the resulting adjustments in their psychological states. These transitions collectively constitute the main source of their psychological stress. A substantial body of empirical research has consistently demonstrated that university students exhibit significantly poorer psychological wellbeing compared to their age-matched counterparts in the general population (Xiong et al., 2020; Kang et al., 2021).

A growing research findings indicates that stress can lead to the development of numerous physical and mental issues in university students. On physical issues, the study focusing on perceived stress levels and dietary patterns among university students reveals a significant correlation: higher perceived stress is associated with poorer dietary habits (Choi, 2020). Furthermore, a study conducted among medical students has confirmed that academic stress can lead to an increase in red blood cells and a decrease in white blood cells in individuals, thereby affecting their overall health (Alhmoud et al., 2021). The most common consequence of stress for university students is sleep problems, such as difficulty falling asleep, frequent nighttime awakenings or nightmares, and other related sleep issues (Li et al., 2024), and this impact is moderate in degree (Schmickler et al., 2023).

Psychological, cognitive, and behavioral issues are also directly impacted by stress. The prediction results of psychological problems are basically consistent across different pressure categories, such as uncertainty stress boosting mental disorder risk in Chinese university students (Wu et al., 2020); adult anxiety risk being linked to early-life stress (Juruena et al., 2020); and interpersonal stress being closely related to teen depression (Belmans et al., 2019). It is worth noting that, the impact of stress on cognition should not be underestimated. An experimental study using brainwave technology has revealed that chronic stress can lead to a decrease in the alertness of attention, as well as impairments in resource allocation and executive functions (Liu et al., 2020). Not only that, the impact of stress is also manifested at the behavioral level. The most common behavioral consequences include impulsive decision-making (Wemm and Wulfert, 2017), unhealthy habits such as excessive eating or alcohol abuse (Böke et al., 2019; Choi, 2020).

Wheatley (1997) presented a “Stress-illness vicious circle” in his early research. In this cycle, all problems are interrelated and affect the entire system, making it impossible for individuals to possess the ability to solve them. Therefore, effective management of stress is extremely necessary. And mindfulness intervention, as a means of stress management, has been extensively studied and proven to be effective.

### 1.2 Mindfulness-based interventions and MBSR

Prior to the 1880s, the term “mindfulness” was utilized in the English language to signify either a specific capacity for focused attention or a positive attribute connected with compassionate consideration for others (Sun, 2014). Mindfulness was formally introduced into the field of empirical medicine alongside the initiation of the Mindfulness-Based Stress Reduction (MBSR) program by Kabat-Zinn (1982) within this program, mindfulness has been employed as a therapeutic intervention for a variety of chronic pain conditions, and it has yielded notable accomplishments in alleviating pain. Since then, mindfulness has drawn the attention of scholars across various disciplines and has gradually been widely adopted (Wang et al., 2021). Kabat-Zinn (2015) regards mindfulness as an openhearted, non-reactive, and non-judgmental awareness cultivated through focusing on the present moment.

As mindfulness interventions extend to broader populations and contexts, their theoretical foundations, operative mechanisms, and functional outcomes have progressively evolved, thereby giving rise to various integrated intervention paradigms. In the realm of mindfulness-based interventions, the most frequently employed approaches encompass Mindfulness-Based Stress Reduction (MBSR), Mindfulness-Based Cognitive Therapy (MBCT), Dialectical behavior therapy (DBT), Relapse prevention (RP) and Acceptance and Commitment Therapy (ACT). However, mindfulness is not the central intervention mechanism for DBT, RP and ACT. Consequently, we shall not delve into it in detail.

In contrast, the practice of mindfulness is more deeply embedded in Mindfulness-Based Stress Reduction (MBSR) and Mindfulness-Based Cognitive Therapy (MBCT), and their therapeutic mechanisms directly rely on the cultivation of mindfulness itself. However, there are significant differences between the two in terms of concepts, core technologies, and theoretical mechanisms.

### 1.3 The emergence of online/digital mindfulness interventions

MBSR is the earliest and most standard form of mindfulness intervention (Nehra et al., 2013). This course encompasses body scanning practices, mindful Hatha Yoga, along with sitting and walking meditation sessions (Santorelli et al., 2017). The course is designed to guide participants in becoming more aware of their physical and mental states, without passing judgment on them. Essentially, it encourages an attitude of acceptance. By doing so, it aids in alleviating participants’ emotional distress, such as stress and anxiety, allowing them to face life’s changes with greater consciousness and flexibility (Kabat-Zinn, 1990; Santorelli et al., 2017). MBCT, it represents an integration of cognitive - behavioral therapy and mindfulness - based stress reduction. This therapeutic approach was originally conceived to mitigate the risk of depression relapse (Segal et al., 2012). MBCT not only incorporates a series of training methods from MBSR, but also emphasizes enhancing metacognitive abilities. That is, by recognizing one’s own thoughts—such as, “This is just an idea; it is not a fact, nor is it the real me”—one can strengthen self-understanding and promote positive self-care (Teasdale et al., 2002; Sipe and Eisendrath, 2012). The preceding discussion has delineated the distinctions between MBSR and MBCT from the perspective of theoretical constructs. Meanwhile, numerous meta-analyses have shown that in clinical populations, studies using mindfulness-based stress reduction as an intervention method are more common (Querstret et al., 2020; González-Martín et al., 2023). Therefore, the results of the intervention studies of MBSR are more likely to be applicable to the university student population.

Additionally, in the realm of empirical research, MBSR course has universal applicability and is not restricted by specific conditions. As it has developed, its format has been diversified and conducted in various autonomous or mixed forms, including online platforms (such as applications, online courses, videos or audio tutorials) as well as books (Hazlett-Stevens and Oren, 2016; Champion et al., 2018; Farris et al., 2021). In contrast, MBCT has a relatively fixed format, requiring highly qualified teachers and emphasizing participant interaction and support (Tickell et al., 2020), which limits its adaptability to other forms. Therefore, the ease of operation and maturity of MBSR make it highly comparable to the traditional form in terms of online implementation.

Based on the above considerations, only mindfulness-based stress reduction (MBSR) was selected as the standardized intervention in this meta-analysis. While traditional mindfulness has demonstrated significant efficacy in managing stress (Cohen-Katz et al., 2005a, 2005b; Frank et al., 2015), its effect sizes vary considerably across different populations (Gawrysiak et al., 2018) and practice dosages (Beblo et al., 2024). However, in light of rapid technological progress and abrupt socio-environmental changes, traditional formats are increasingly seen as insufficient (Smith, 2014; Mani et al., 2015). This has led to the emergence of online mindfulness programs. And the stress-reduction effects of these evolving digital interventions continue to show heterogeneity across modalities (Wolever et al., 2022).

### 1.4 Gaps in the literature and aims of the present meta-analysis

In the previous meta-analysis on the impact of mindfulness intervention on the stress of university students, the relevant studies mainly focused on the following aspects: (a) Healthcare students are the main research objects (Hathaisaard et al., 2022; Sperling et al., 2023; da Silva et al., 2023; Karo et al., 2024). (b) In the analysis of the implementation methods of mindfulness interventions, the latest meta-analysis has mainly focused on the single online approach (Sommers-Spijkerman et al., 2021; Gong et al., 2023) and the combined approach of online and traditional methods (Hathaisaard et al., 2022; Sperling et al., 2023; da Silva et al., 2023; Karo et al., 2024). However, in the meta-analysis of the combination of online and traditional methods, most of the studies did not strictly distinguish between these two forms. And, the results did not report subgroup analyses conducted according to implementation method (online mindfulness vs. traditional mindfulness). (c) Most of the current meta-analyses treat various mindfulness paradigms (such as MBSR, MBCT, ACT) as a whole for research, without making specific distinctions (Sommers-Spijkerman et al., 2021; Hathaisaard et al., 2022; Sperling et al., 2023; Gong et al., 2023; Karo et al., 2024).

Several recent systematic reviews have synthesized evidence on digital mental health interventions for students. For example, a review by Alrashdi et al. (2024) focused specifically on the efficacy and attrition rates of online mindfulness-based interventions for university students’ psychological distress and wellbeing. While such reviews are valuable, key gaps remain. The present meta-analysis extends this literature in three specific ways: (1) it provides a focused, comparative analysis by including both online and traditional (face-to-face) mindfulness-based interventions within the same review, allowing for an indirect comparison of their efficacy; (2) it adopts a targeted focus on stress as a primary, well-defined outcome; and (3) it conducts exploratory analyses of implementation characteristics (e.g., duration, session frequency, delivery platform) that may influence effectiveness, which can inform practical application. By addressing these aspects, this review aims to offer more nuanced evidence to guide the selection and design of mindfulness programs in higher education settings.

Therefore, it is necessary to compare the effects of online mindfulness and traditional mindfulness on stress mitigation, and explore effective strategies to adapt to dynamically changing needs in university students. It is important to note that this review compares online and traditional formats indirectly, by synthesizing trials that separately compare each format against a control condition, as head-to-head RCTs directly comparing both formats within the same study were not eligible for inclusion.

## 2 Methods

The current study was registered in PROSPERO (registration number: CRD420251025123) and followed the Preferred Reporting Items for Systematic Reviews and Meta-Analyses (PRISMA) guidelines (Moher et al., 2009; Page et al., 2021).

### 2.1 Sources and search strategy

The selection of Web of Science, Embase, Medline and PsycINFO as primary data sources was justified by their prolonged temporal span, multidisciplinary coverage, and regional diversity (Bramer et al., 2017). Based on the following considerations, articles published between January 1, 2010 and June 1, 2025 were selected for the literature search. First, the marked increase in publication volume after 2010, combined with the standardization of research methodologies, significantly improved the quality of empirical evidence in this field (Baminiwatta and Solangaarachchi, 2021). Then, this period witnessed rapid expansion of online health applications, paralleling the widespread adoption of mobile internet technologies and smart devices (Marsano et al., 2015; Firth et al., 2016; Larsen et al., 2016). In addition, these years encompass the expansion of mindfulness-based interventions were applied beyond clinical settings to areas such as emotion regulation and workplace wellbeing (Zenner et al., 2014; Lomas et al., 2017).

Before initiating the systematic literature search, three authors (YW, ZP and YH) independently screened existing publications on online mindfulness interventions. We have defined and classified online mindfulness interventions according to their core implementation techniques. The final search index word was established through an iterative process that involved team discussion and multiple preliminary search tests. The complete search term and syntax rules are documented in Appendix 1. Literature retrieval was restricted exclusively to English-language publications across all data sources. In addition, records were excluded if they were review articles, conference proceedings, meeting abstracts, book reviews, news items, letters, or similar publication types.

Following the predefined PICOS criteria and adopting a double-blind review process, two authors (YW and ZP) independently screened articles based on titles, keywords, abstracts, and full texts. Firstly, the two aforementioned authors completed preliminary screening independently and simultaneously, recording decisions separately. Subsequently, they performed article inclusion/exclusion procedures. If their decisions conflicted, the disputed articles were submitted to a third author (WQ) for arbitration based on the predefined PICOS criteria.

Due to resource constraints for translation, the literature search was restricted to English-language publications. Grey literature (e.g., theses, conference proceedings) and trial registries were not systematically searched.

### 2.2 Eligibility criteria

The study applied the PICOS framework to establish eligibility and criteria, following the data collection criteria from the Cochrane Handbook for Systematic Reviews of Interventions (Higgins and Green, 2011). The inclusion criteria include: (a) P-Participants. The study population was restricted to university students. (b) I-Intervention. Online mindfulness or traditional mindfulness shall serve as the sole intervening measure. And, the specific strategies of online mindfulness comprise, but are not limited to, app-based mindfulness, VR-based mindfulness and online teaching-based mindfulness. The crucial requirement is that all intervention strategies are implemented within virtual environments. Traditional mindfulness is operationally defined as mainly based on offline intervention, including implementation based on individuals and groups. All mindfulness interventions followed either the standard MBSR protocol developed by Kabat-Zinn or adapted its foundational principles. (c) C-Comparison. Participants in the control group were not given any intervention, enabling extraction of non-intervention data for analysis. (d) O-Outcome. The change in stress levels pre-post intervention was the outcome, measured with standardized scales such as the Perceived Stress Scale (PSS). (e) S-Study design. Included studies exclusively employed randomized controlled trial designs. And the exclusion criteria include: (a) P-Participants. The study population comprised non-collegiate individuals. (b) I-Intervention. The intervention group received either blended mindfulness approaches (e.g., offline mindfulness and online mindfulness) or non-mindfulness interventions (e.g., exercise, yoga). (c) Participants in the control group received other interventions (e.g., yoga). (d) O-Outcome. Changes in stress outcomes were measured using alternative indicators, including qualitative or physiological parameters. (e) S-Study design. Excluded studies employed non-rct studies (e.g., cohort studies, case reports).

The primary outcome was the change in stress levels pre-post intervention. The secondary outcome was the change in mindfulness levels. “Online mindfulness intervention(s)” is our preferred term for any mindfulness-based program delivered primarily through digital means (apps, websites, audio/video). “Traditional mindfulness intervention(s)” is our preferred term for any mindfulness-based program delivered primarily face-to-face, in individual or group settings. We consistently refer to the primary outcome as “stress” (e.g., “stress levels,” “stress reduction”). We specify in the Methods (2.2, O-Outcome) that this was operationalized as scores on standardized scales, most commonly the Perceived Stress Scale (PSS). We consistently refer to the secondary outcome as “mindfulness” (e.g., “mindfulness levels,” “mindfulness outcomes”).

This study employed EndNote 20 for literature screening and management. This study employed EndNote 20 for literature screening and management. All identified records will be screened at multiple levels according to PRISMA guideline. The systematic screening process includes: database retrieval, deduplication, hierarchical screening, and final inclusion.

The text now more explicitly states that studies were included if they evaluated either an online or a traditional mindfulness intervention against a control, and that studies containing both formats (direct comparators) were excluded per our criteria. Interventions were those described as mindfulness-based and incorporating core MBSR elements (e.g., body scan, mindful breathing), even if adapted or delivered digitally.

Eligible trials were those that randomized participants to either an online mindfulness intervention arm or a traditional (face-to-face) mindfulness intervention arm, each compared against a passive control group (e.g., waitlist, treatment-as-usual, no intervention). Studies containing both online and traditional mindfulness arms within the same trial (head-to-head comparisons) were excluded to maintain a clear contrast against a common control condition for the indirect comparison.

Included interventions were those described by the study authors as ‘mindfulness-based’ and incorporating core foundational principles and practices derived from Mindfulness-Based Stress Reduction (MBSR). These core components typically included: formal mindfulness practice (e.g., focused attention on breath, body scan), psychoeducation about stress and mindfulness, and an emphasis on present-moment awareness without judgment. Programs were included even if they were delivered in adapted (e.g., abbreviated duration, digital format, integrated with compassion elements) or self-guided formats, provided their primary therapeutic mechanism was explicitly framed as mindfulness training.

### 2.3 Data extraction

Two authors (YW and YH) independently extracted data in accordance with the Cochrane Handbook for Systematic Reviews of Interventions, accounting for planned subgroup analyses. The extracted data encompassed: article characteristics, participant demographics, task specifications, sample sizes of intervention and control groups, and other relevant information. Following independent extraction, the authors cross-verified all entries. A third author (HL) served as arbitrator to resolve discrepancies through consensus or final adjudication. This process was implemented using Microsoft Excel, as demonstrated in the section on the characteristics of included studies.

### 2.4 Quality assessment

Two authors (YW and HL) independently assessed the risk of bias and certainty of evidence in the included articles, with any discrepancies resolved through discussion with a third author (WQ). The Cochrane Risk of Bias 2.0 (RoB 2) tool, an authoritative method for assessing risk of bias in randomized controlled trials, it covers the following five domains: the randomization process, deviations from intended interventions, missing outcome data, measurement of the outcomes, and selection of the reported results. Each domain includes several signaling questions. The risk of bias for these five domains will be classified into three levels (low risk of bias, some concerns, and high risk of bias) through systematic examination. Ultimately, the overall risk of bias for an article will be determined based on the comprehensive assessment (Chandler et al., 2019).

### 2.5 Data synthesis and statistical analysis

Analyses were conducted separately for the primary outcome (stress) and the secondary outcome (mindfulness). This meta-analysis involved parallel-group randomized controlled designs. The standardized mean difference (SMD) was chosen as the effect size indicator, thereby enabling the comparability of research results from different measurement tools or units (Borenstein et al., 2021). According to Cohen’s criteria (Cohen, 2013), effect sizes are classified as small effect (0.2), medium effect (0.5), and large effect (0.8). To calculate the effect size for each study, the sample sizes, means, and standard deviations before and after the intervention were selected for continuous variables. For those who need to combine subgroup data for research purposes, the calculation should be carried out according to the combined group formula provided by Cochrane (Chandler et al., 2019). Combined sample size formula: *N=N_1_ + N_2_*; Combined mean formula: *M=* $\frac{N1M1+N2M2}{N1+N2}$ ; Combined standard deviation formula:


$$\mathrm{SD}=\sqrt{\frac{\left(N_{1}-1)SD_{1}^{2}+(N_{2}-1)SD_{2}^{2}+\frac{N_{1}N_{2}}{N_{1}+N_{2}}(M_{1}^{2}+M_{2}^{2}-2M_{1}M_{2}\right)}{N_{1}+N_{2}-1}}$$

According to the guidance in the Cochrane Handbook (Chandler et al., 2019), when the results show low heterogeneity (I^2^ < 25%), the subsequent analysis adopts the fixed effect model. If the results of the random effect model are similar to those of the fixed effect model at this time, it can further support this choice.

As part of heterogeneity assessment, the Q statistic was used for testing heterogeneity, and the I^2^ statistic was used for quantifying the proportion of variance. The former is used to test the significance of heterogeneity, with *p* < 0.05 indicating the presence of heterogeneity; the latter is used to test the magnitude of heterogeneity. The larger the I2 value, the greater the heterogeneity (Chandler et al., 2019). For the heterogeneous results where I^2^ ≥ 50%, the sources of heterogeneity will be further investigated.

The study conducted a sensitivity analysis using the elimination-by-omission method, with the aim of assessing the robustness of the combined effect size. The stability of the effect size and significance of statistical were assessed by examining the magnitude of change in the point estimate and whether the 95% confidence interval straddled the invalid values (Borenstein et al., 2021).

To assess publication bias, objective methods including a funnel plot and Egger’s test were employed, minimizing subjective evaluation. If the *p*-value of the Egger test is greater than 0.05, it suggests no significant evidence of publication bias, and the research results may be considered more robust (Borenstein et al., 2021).

All the above analyses were conducted using Stata (version 17). A sensitivity analysis excluded studies with a high risk of bias to the Methods. For multi-arm trials, relevant intervention arms were combined using Cochrane formulas to avoid double-counting control participants.

Hedges’ g was calculated as the effect size measure to adjust for small-sample bias (Borenstein et al., 2021). Effect sizes were computed using post-intervention means and standard deviations for the intervention and control groups. When only change score means and standard deviations were reported, the post-intervention standard deviation was reconstructed using the Cochrane Handbook formula (Chandler et al., 2019), assuming a pre-post correlation of r = 0.7, based on recommendations for psychological interventions (Furukawa et al., 2021). This strategy (preferring post-intervention scores, reconstructing SDs when necessary with a consistent correlation) was applied uniformly across all studies to ensure comparability.

For the two included three-arm trials (Djernis et al., 2021; Dark-Freudeman et al., 2020), relevant mindfulness-based intervention arms were combined into a single intervention group using the Cochrane formulas for combining groups provided above (Chandler et al., 2019), prior to calculating the effect size versus the shared control group. This approach avoided double-counting participants in the control group and ensured each study contributed one independent comparison to the meta-analysis. A random-effects model was used for all meta-analyses to account for expected heterogeneity across studies. A fixed-effect model was considered only when heterogeneity was negligible (I^2^ < 25%) and the pooled estimate was similar between models.

A sensitivity analysis was conducted by excluding studies judged to be at a high overall risk of bias.

## 3 Results

### 3.1 Study selection

**Figure 1:**
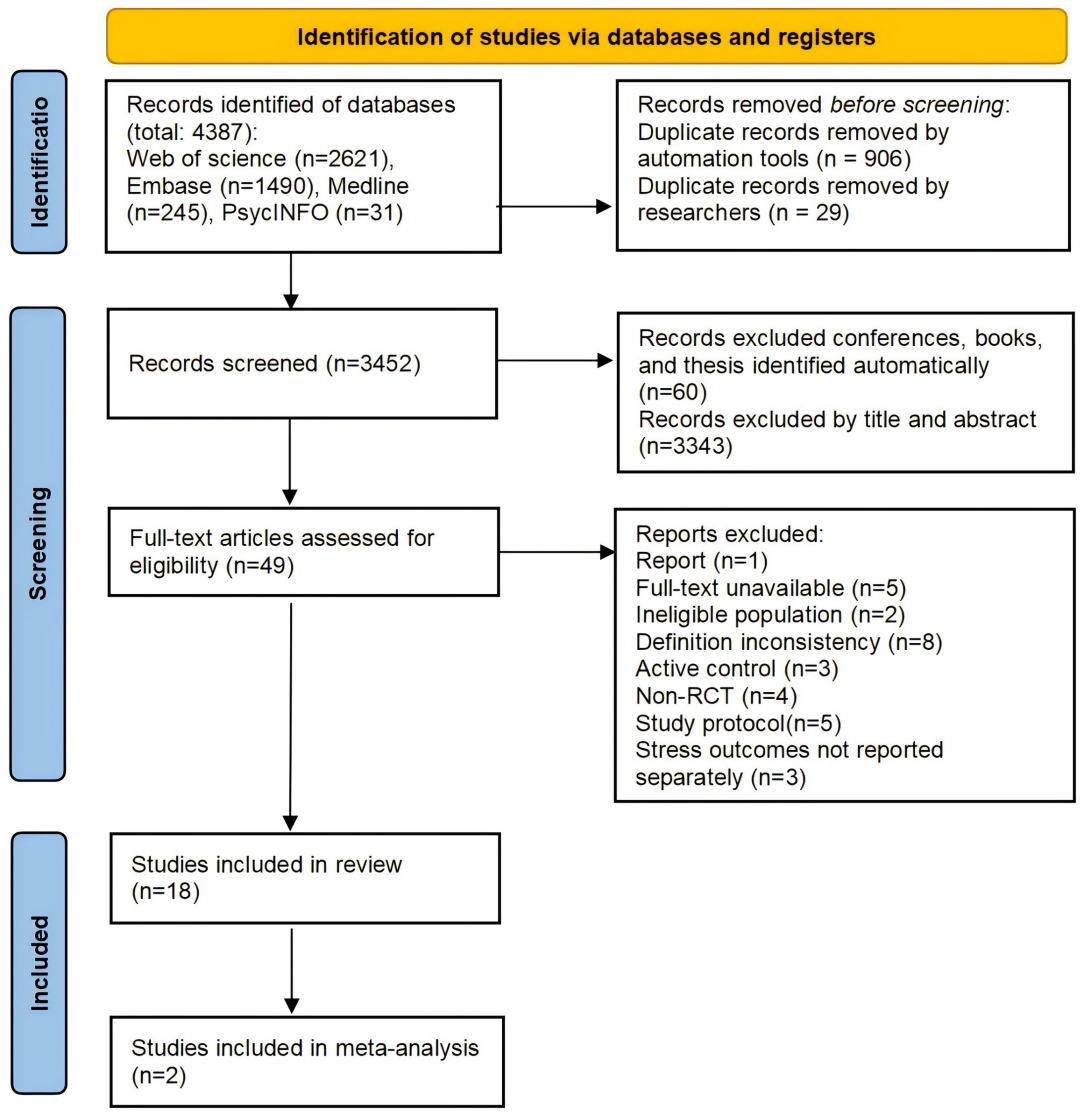
PRISMA flow chart for study selection.

**Figure 2:**
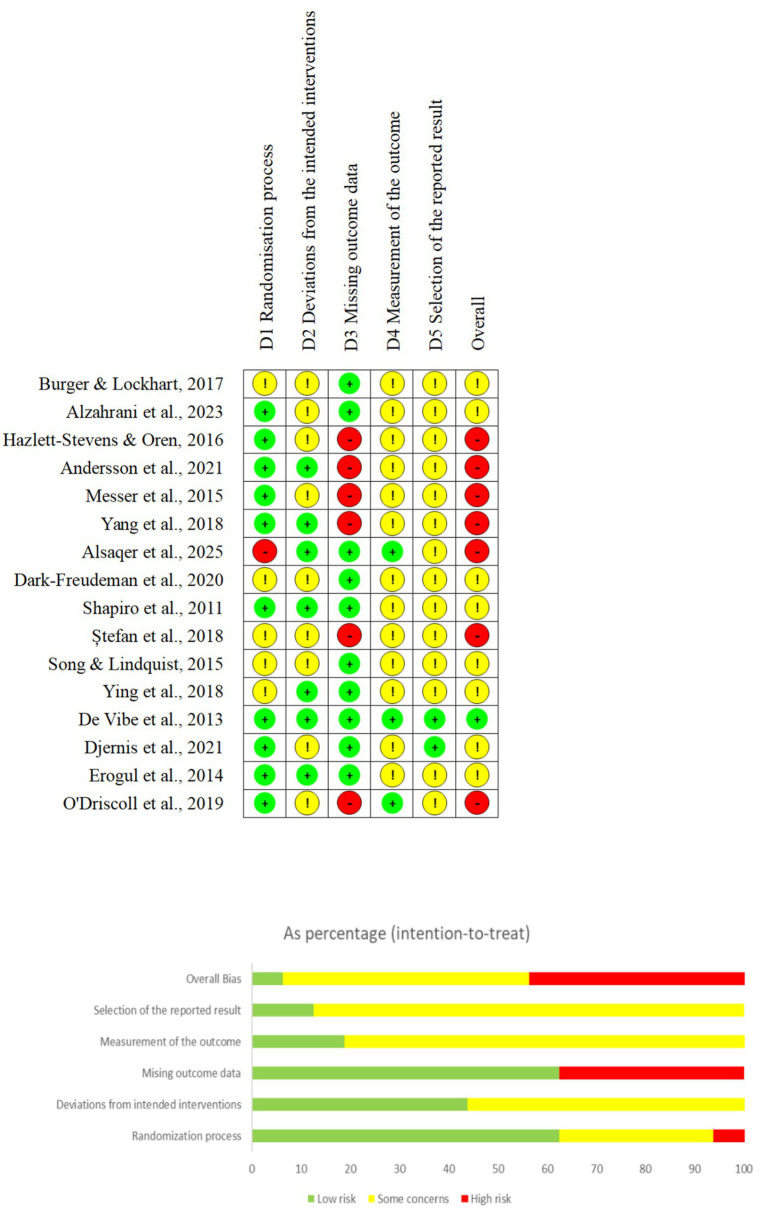
Risk of bias across studies.

A total of 4,387 studies were identified through the specified sources and search strategy (see Figure 1). Among these, 935 duplicate records were removed by automated tools (*n =* 906) and researchers (*n =* 29). Subsequently, records pertaining to conferences, books, and thesis were excluded automatically (*n =* 60) and further exclusions were made after manual screening of titles and abstracts (*n =* 3,343). A total of 49 articles underwent full-text examined. Of these, 18 studies satisfied the predefined inclusion criteria for systematic review. However, 2 study was subsequently excluded due to unsuccessful data acquisition attempts (no response from corresponding authors), resulting in a final meta-analysis of 16 studies (Figure 2).

### 3.2 Overview of study characteristics

All the details from 16 studies are demonstrated in Table 1. Among the included studies, six investigated online mindfulness interventions (Burger and Lockhart, 2017; Alzahrani et al., 2023; Hazlett-Stevens and Oren, 2016; Andersson et al., 2021; Messer et al., 2015; Yang et al., 2018), while the other 10 focused on traditional mindfulness interventions (Alsaqer et al., 2025; Dark-Freudeman et al., 2020; Shapiro et al., 2011; Ștefan et al., 2018; Song and Lindquist, 2015; Ying et al., 2018; De Vibe et al., 2013; Djernis et al., 2021; Erogul et al., 2014; O'Driscoll et al., 2019). Overall, the majority of studies (71.4%) were conducted in Western countries.

**Table 1:** Characteristics of the selected studies.

Characteristics of the article		Characteristics of the participants			Intervention group vs. control group (*n*)		Attrition (%)	Intention-to-treat	Control condition	Task information		Measure (type of assessment)	The longest follow-up	Result
Author and year	Country	Age	Gender (*n*)	Population	Baseline	End point				Strategy	Treatment duration; frequency			
Burger and Lockhart (2017)	United States	18–20 years: *n =* 36	Male:9 Female:43	Prelicensure nursing students	32/28	28/24	13.3	No	Control group with no intervention	Online instruction on MM techniques (via audio file)	Duration: 4 weeks Frequency: 10 min practice daily	Perceived Stress Scale (PSS-10) The Five-Facet Mindfulness Questionnaire (FFMQ-39)	No	The meditation group showed significantly lower post-intervention stress compared to controls.
Alzahrani et al. (2023)	Saudi Arabia	IG: Mea*n =* 22.15 WLC: Mea*n =* 22.44	Male:30 Female:54	Medical students	39/45	29/42	15.4	No	Wait-list Control	Mindfulness-based Stress Reduction with audiovisual materials	Duration: 8 weeks Frequency: 2.5 h of sessions per week	Perceived Stress Scale (PSS-10) The Mindful Attention Awareness Scale (MAAS-15)	3-month	The MBSR group had a slightly lower PSS score
Hazlett-Stevens and Oren (2016)	United States	Mea*n =* 22.10	Male:16 Female:51 Did not specify: 1	Undergraduate and graduate students	47/45	25/43	26.1	No	Control group with no intervention	Self-help bibliotherapy format of the MBSR intervention	Duration: 10 weeks Frequency: read one chapter per week and use the mp3 recordings	Perceived Stress Scale (PSS-10) Depression Anxiety Stress Scales (DASS-21) The Five-Facet Mindfulness Questionnaire (FFMQ-39)	No	No significant time x group interaction effect was found for perceived stress between the bibliotherapy and control groups
Andersson et al. (2021)	Sweden	Mea*n =* 25	Male:17 Female:39 Did not specify: 1	University students	25/15	25/15	12.3	ITT	Wait-list Control	Mindfulness training program via a mindfulness app.	Duration: 6 weeks Frequency: 7 days introductory course, self-administered practice of 5 weeks (10–15 min per day)	Perceived Stress Scale (PSS-10)	No	Mindfulness training did not show a significant reduction in stress to the waitlist control group
Messer et al. (2015)	United States	No report	Male:52 Female: 105	University Students	53/52	23/49	27	No	Control group with no intervention	Mindfulness training via audio clips	Duration: 3 weeks Frequency: at least three different days during the week (12–20 min per day)	Perceived Stress Scale (PSS-14) The Five-Facet Mindfulness Questionnaire (FFMQ-39)	No	Mindfulness conditions experienced lower stress than the control condition
Yang et al. (2018)	United States	Mea*n =* 25.11	Male: 32 Female: 56	Medical students	45/43	40/40	9.1	ITT	Wait-list Control	An audio-guided mindfulness meditation programme via a mobile application Headspace	Duration: 30 days Frequency: 10 min for the first 10 days, 15 min for the next 15 days, and 20 min for all subsequent sessions	Perceived Stress Scale (PSS-10) The Five-Facet Mindfulness Questionnaire (FFMQ-39)	60-day	Perceived stress significantly decreased for the intervention group
Alsaqer et al. (2025)	Jordan	IG: Mea*n =* 21.4 WLC: Mea*n =* 21.6	Male: 33 Female: 61	Undergraduate Nursing Students	52/52	52/52	0	No	Wait-list Control	Mindfulness-Based Stress Reduction	Duration: 6 weeks Frequency: once-a-week sessions for 2 h, and home practice freely	Depression Anxiety Stress Scales (DASS-21) The Mindful Attention Awareness Scale (MAAS-15)	No	MBSR students reported significantly greater decreases in stress
Dark-Freudeman et al. (2020)	USA	Mea*n =* 20.92	Male: 30 Female: 47	Undergraduate students	30/30	23/28	12.5	No	Control group with no intervention	Mindfulness-Based Intervention (MBI)	Duration: 4 weeks Frequency: small-group intervention sessions weekly for 30 min, and practice listening to the traditional MBI technique for 15 min daily	Perceived Stress Scale (PSS-10)	No	Perceived stress scores significantly decreased among the MBI group
Shapiro et al. (2011)	United States	Mea*n =* 18.73	Male: 4 Female: 26	University students	15/15	15/15	0	No	Wait-list Control	Mindfulness-Based Stress Reduction (MBSR)	Duration: 8 weeks	Perceived Stress Scale (PSS-10) The Mindful Attention Awareness Scale (MAAS-15)	2-month 12-month	MBSR participants, relative to controls, reported marginally larger declines in PSS stress
Ștefan et al. (2018)	Romania	Mea*n =* 18.92	Male: 5 Female: 66	College Students at Risk for Social Anxiety	36/35	22/24	35.2	No	Wait-list Control	Mindfulness-Based Stress Reduction (MBSR)	Duration: 6 weeks Frequency: one group session per week lasting 1.5–2 h and daily home practice via the Facebook group for 30 min	Perceived Stress Scale (PSS-10)	No	MBSR participants reported significantly less perceived stress compared to the control group
Song and Lindquist (2015)	South Korea	Mea*n =* 19.6	Male: 8 Female: 36	Nursing students	25/25	21/23	12	No	Wait-list Control	Mindfulness-Based Stress Reduction (MBSR)	Duration: 8 weeks Frequency: 2 h per week and home practice	Depression Anxiety Stress Scales (DASS-21) The Mindful Attention Awareness Scale Korean version (MAAS-20)	No	Compared with WL participants, MBSR participants reported significantly greater decreases in stress
Ying et al. (2018)	China	Mea*n =* 24.31	Male: 16 Female: 22	College students	20/18	20/18	0	No	Control group with no intervention	Mindfulness-Based Stress Reduction (MBSR)	Duration: 8 weeks Frequency: one group session per week and home practice freely	Perceived Stress Scale (PSS)	No	Mindfulness training intervention program is effective in reducing students’ academic stress
De Vibe et al. (2013)	Norway	Mea*n =* 23.8	Male: 69 Female: 219	Medical and psychology students	144/144	144/144	3.8	ITT	Control group with no intervention	Mindfulness-Based Stress Reduction (MBSR) programme	Duration: 7 weeks Frequency: six weekly sessions of 1.5 h each, a 6-h session in week seven, and 30 min of daily home mindfulness practice	Perceived Medical School Stress (PMSS-13) The Five-Facet Mindfulness Questionnaire (FFMQ-39)	No	A mindfulness intervention can reducemental study stress
Djernis et al. (2021)	Denmark	Mea*n =* 30.6	Male: 8 Female: 52	Bachelor’s or master’s degree students	42/18	38/17	8.3	No	Control group with no intervention	Residential mindfulness programme based on the 8-week MBSR programme	Duration: 5 days Frequency: 6–8 h per day	Perceived Stress Scale (PSS-10) The Five-Facet Mindfulness Questionnaire (FFMQ-39)	3-month	Stress decreased with small to medium effect sizes post-intervention, although not statistically significant.
Erogul et al. (2014)	United States	Mea*n =* 23.5	Male: 31 Female: 26	First year medical students	29/30	28/30	1.7	No	Control group with no intervention	Eight-week abridged Mindfulness-Based Stress Reduction (MBSR) program	Duration: 8 weeks Frequency: group instruction for 75 min, once per week, for 8 weeks supplemented by a program of suggested meditation at home. Between the 7th and 8th weekly meeting, students attended a full day retreat offsite	Perceived Stress Scale (PSS-10)	6-month	The treatment group achieved significant reduction on PSS scores at the conclusion of the study
O'Driscoll et al. (2019)	Ireland	18–20 years: *n =* 62 21–23 years: *n =* 26 ≥ 24 years: *n =* 11	Male: 33 Female: 66	Undergraduate pharmacy students	81/83	51/48	40	No	Wait-list Control	Based on the principles underpinning Doctor Jon Kabat-Zinn’s 8 week MBSR course	Duration: 4 weeks Frequency: weekly sessions of 1.5 h and practice daily for 20 min.	Perceived Stress Scale (PSS-10) The Five-Facet Mindfulness Questionnaire (FFMQ-39)	No	While stress scores showed improvements, these results were not statistically significant.

A total of 1,393 participants were enrolled in this study, comprising 715 individuals in the intervention group, 678 in the control group. Among the studies included in the analysis, except for Messer et al. (2015), which did not report the age of the participants, other studies reported age information in different ways. One reported the age range of some participants (Burger and Lockhart, 2017), whereas the remaining studies either provided the average age of the participants or indicated the number of participants within a specific age range. In terms of gender composition, the study comprised 393 male participants, 969 female participants, and 2 participants whose gender was not specified. Regarding the characteristics of the participants, the participants were general undergraduate students in 7 of the studies, and one study involved students with a propensity for social anxiety (Ștefan et al., 2018). The other eight studies were all conducted with students majoring in medical nursing.

The dropout rates in these studies ranged from 0 to 40%, of which three applied intention-to-treat (ITT) analysis (Messer et al., 2015; Alsaqer et al., 2025; De Vibe et al., 2013). Moreover, certain methodological aspects require comprehensive clarification. The present analysis included 14 two-arm studies and 2 three-arm studies with varying control group arrangements. Specifically, one study (Djernis et al., 2021) set up two groups of mindfulness-based interventions in different environments (an indoor mindfulness group and an outdoor mindfulness group), along with a control group on a waiting list. Another study (Dark-Freudeman et al., 2020) adopted a three - arm design comprising a mindfulness intervention group, a coloring intervention group, and a traditional control group. Based on research objectives and consideration of experimental group independence in study designs, relevant experimental and control groups were systematically selected across studies for comprehensive analysis. Intervention durations ranged from 5 days to 10 weeks across studies. Additionally, most studies reported specific delivery methods, implementation frequency, and adherence with home practice. Five of the included studies implemented follow-up evaluations (Alzahrani et al., 2023; Yang et al., 2018; Shapiro et al., 2011; Djernis et al., 2021; Erogul et al., 2014). Apart from these, one study (Shapiro et al., 2011) included two follow - up assessments, conducted separately at 2 months and 12 months. Stress outcomes served as endpoints across all included studies. As for the results of mindfulness, 11 studies have reported the relevant findings of mindfulness (Burger and Lockhart, 2017; Alzahrani et al., 2023; Hazlett-Stevens and Oren, 2016; Messer et al., 2015; Yang et al., 2018; Alsaqer et al., 2025; Shapiro et al., 2011; Song and Lindquist, 2015; De Vibe et al., 2013; Djernis et al., 2021; O'Driscoll et al., 2019).

### 3.3 Risk of bias assessment

The risk of bias in the included studies was assessed using the Cochrane RoB 2 tool. The comprehensive assessment results show that, given the rigorous description and implementation process of the research methodology, the overall risk of bias for one of the studies was assessed as low (De Vibe et al., 2013).

For the remaining eight studies (Burger and Lockhart, 2017; Alzahrani et al., 2023; Dark-Freudeman et al., 2020; Shapiro et al., 2011; Song and Lindquist, 2015; Ying et al., 2018; Djernis et al., 2021; Erogul et al., 2014), since at least one quality assessment domain was rated as having “some concerns,” their overall risk of bias was also judged to be of “some concern.” In the evaluation of the randomization process, these studies (Burger and Lockhart, 2017; Dark-Freudeman et al., 2020; Song and Lindquist, 2015; Ying et al., 2018) were rated as having “some concerns” because they specified the use of random allocation but did not report whether double-blinding was implemented. In the evaluation of the deviations from intended interventions, although dropout rates exceeded 5% in these studies (Burger and Lockhart, 2017; Alzahrani et al., 2023; Dark-Freudeman et al., 2020; Song and Lindquist, 2015; Djernis et al., 2021), assessment of whether withdrawal reasons were intervention-related (e.g., perceived lack of efficacy or adverse effects) is essential for bias evaluation. All studies documented participant withdrawal reasons, primarily time constraints, religious beliefs, or non-completion of post-intervention questionnaires. There is no evidence to confirm the problem of missing cases bias. Therefore, the evaluation of these studies in this field is “some concerns.” In the evaluation of the missing outcome data, these 8 studies all appropriately addressed missing data, and were therefore judged to be at low risk of bias. In the evaluation of the measurement of the outcome, although these studies did not blind assessors, per RoB 2 criteria: “Knowledge of the intervention received could have influenced outcome assessment, but there is no evidence that it actually did,” they were assessed as having “some concerns.” In the evaluation of the selection of the reported result, only study (Djernis et al., 2021) had a preregistered protocol; the remaining studies were assessed solely for trial registration completeness, leading to a “some concerns” rating.

The overall evaluation of the seven studies is high risk (if there is high risk in any one area, the overall risk is high). Among them, Alsaqer et al. (2025) was rated as high risk because there was no blinding in the participant allocation stage; the remaining 6 studies, although they contained two ITT analyses (Andersson et al., 2021; Yang et al., 2018), they still belong to high risk because the reasons for missing data were not explained in all of them.

## 4 Meta-analytic results

### 4.1 Stress outcome (primary)

#### 4.1.1 Exploratory subgroup analyses overall effects of mindfulness on stress by student type

We now explicitly present the primary random-effects meta-analysis including all 16 studies upfront. We report its pooled effect size, confidence interval, and the associated high heterogeneity (e.g., SMD = −0.58, 95% CI: −0.78 to −0.38, I^2^ = 80.35%). This provides the full, unadjusted picture of the evidence. We then clearly frame the removal of outliers (Ying et al., 2018; De Vibe et al., 2013) as a pre-planned sensitivity analysis aimed at exploring sources of heterogeneity. We report the results of this trimmed analysis separately (e.g., SMD = −0.60, 95% CI: −0.74 to −0.47, I^2^ = 15.58%).

**Figure 3:**
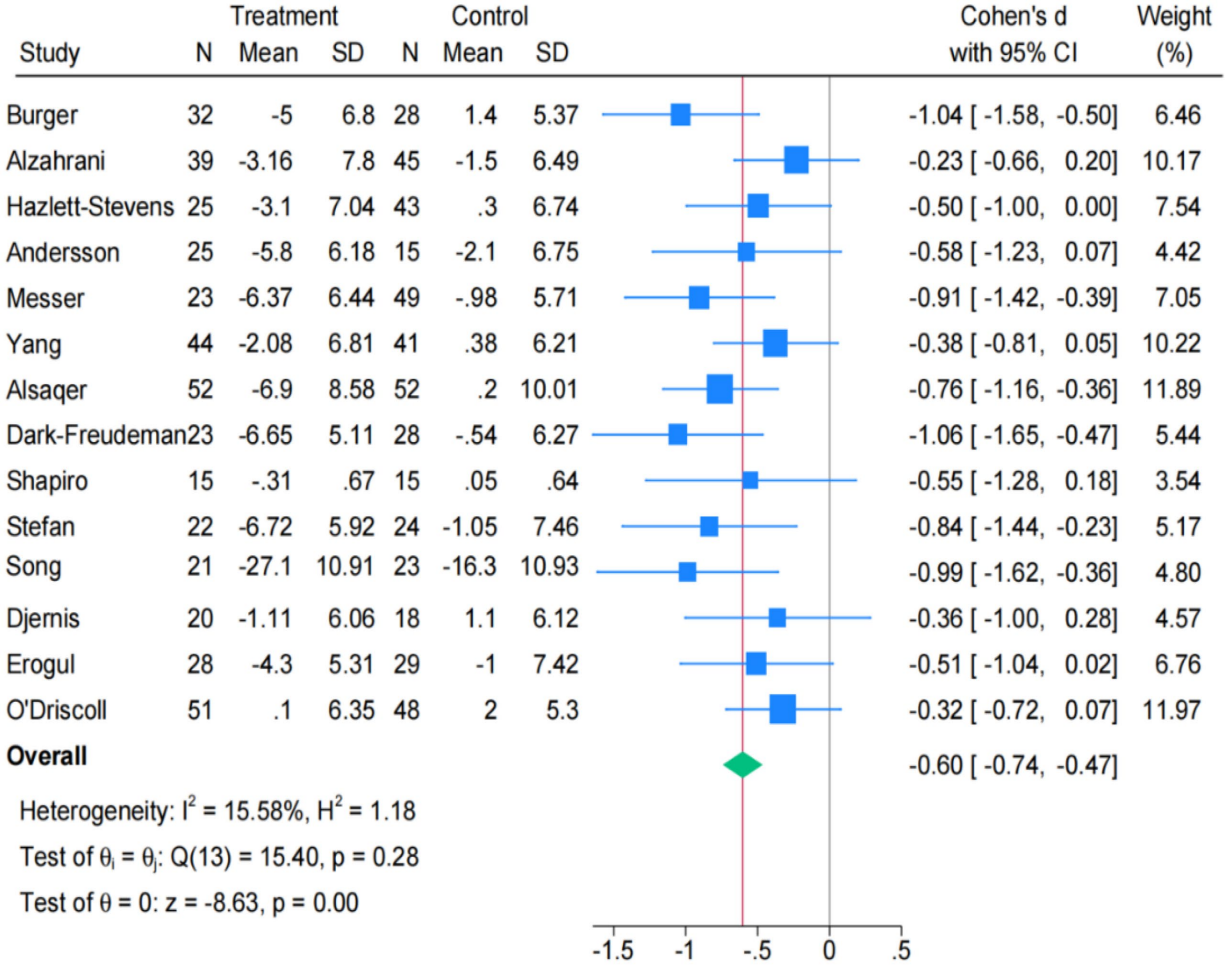
Forest plot for stress (*k* = 14).

All the studies included above evaluated stress. Firstly, the study comprehensively examined the overall impact of online mindfulness and traditional mindfulness on stress, and found a significant degree of heterogeneity [Q(15) = 76.34, *p* < 0.001; I^2^ = 80.35%] (Appendix 2). Using the Galbraith plot to conduct a more precise study on high heterogeneity, the results showed that there was a significant degree of heterogeneity in the Ying et al. (2018) and De Vibe et al. (2013) studies (Appendix 3). After eliminating the above two studies one by one, the heterogeneity decreased from I^2^ = 80.35% to I^2^ = 43.37% (Appendix 4), and the final result showed low heterogeneity Q(13) = 15.40, *p* = 0.28, I^2^ = 15.58% (Figure 3). Online and traditional mindfulness interventions both significantly reduce stress levels in university students. The pooled effect size is SMD = −0.60 (95% CI: −0.74 to −0.47), and the intervention effect has a highly significant statistical meaning (z = −8.63, *p* < 0.001).

#### 4.1.2 Exploratory subgroup analyses of effects of mindfulness on stress by student type

**Figure 4:**
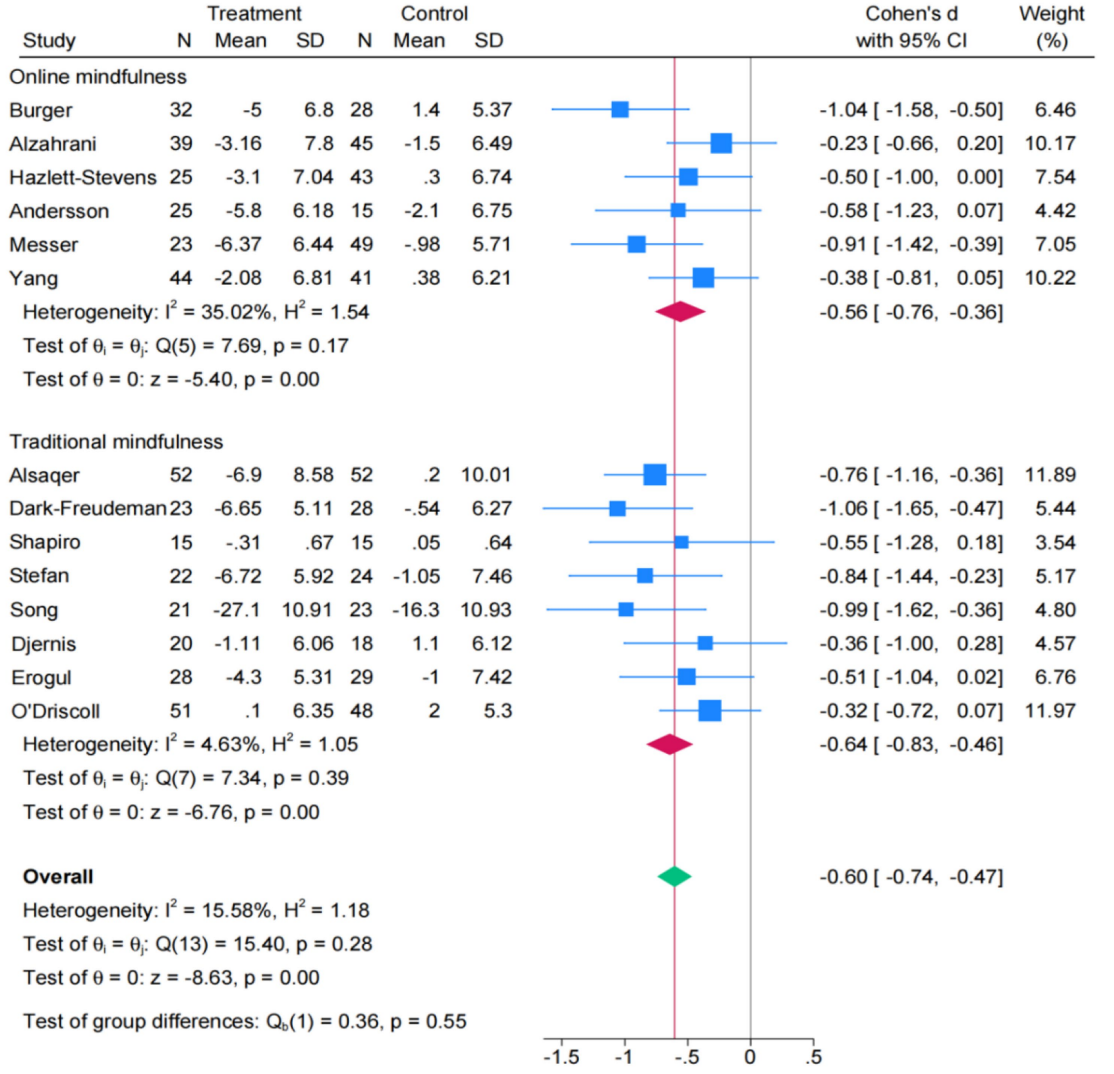
Forest plot of subgroup for stress (*k* = 14).

In order to examine differences in the impact of online vs. traditional mindfulness interventions on stress, a further subgroup analysis will be conducted next (Figure 4). The results indicate that both online mindfulness interventions (SMD = −0.56; 95% CI: −0.76, −0.36) and traditional mindfulness interventions (SMD = −0.64; 95% CI: −0.83, −0.46) effectively reduced stress, with comparable effect sizes. It is indicated that both intervention models have a medium effect size on reducing stress levels. Furthermore, there was no statistically significant difference (*p* = 0.55) in the effect sizes between the two intervention methods, indicating that the effects of the two intervention approaches were comparable.

#### 4.1.3 Exploratory subgroup analyses of effects of mindfulness on stress by student type

Participants were stratified into two cohorts: general students and healthcare students. Intervention effects on stress were analyzed by delivery modality (online vs. traditional) to identify differential responsiveness across student subgroups (Appendix 5).

The results of the study show that for general students (Table 2), online mindfulness interventions showed an effective stress-reducing effect (SMD = −0.67; 95% CI: −0.98, −0.35), with traditional interventions yielding similar results (SMD = −0.73; 95% CI: −1.05, −0.41). This indicates that both online and traditional mindfulness interventions have a moderate level impact on the stress experienced by the general student population, and the effects of the two methods are comparable (*p* = 0.781). For healthcare students, online mindfulness interventions demonstrated SMD = −0.48 (95% CI: −0.75, −0.22), and traditional interventions showed SMD = −0.60 (95% CI: −0.83, −0.37). The results show that both the online and traditional mindfulness intervention methods have a moderate impact on the stress levels of the healthcare workforce, and the effects of these two methods are comparable (*p* = 0.519).

**Table 2:** Summary of intervention effects on stress in student subgroups.

Variable	Subgroup	Studies (*N*)	Sample size	SMD [95% CI]	Two-tailed test	
					Z	*P*
General students	Online mindfulness	3	180	−0.67 [−0.98, −0.35]	−4.15	0
	Traditional mindfulness	4	165	−0.73 [−1.05, −0.41]	−4.52	0
Healthcare students	Online mindfulness	3	229	−0.48 [−0.75, −0.22]	−3.56	0
	Traditional mindfulness	4	304	−0.60 [−0.83, −0.37]	−5.07	0

#### 4.1.4 Exploratory analyses of effects of mindfulness on stress in delivery-mode subgroups by delivery mode

Subgroup analyses confirmed comparable stress reduction effects between online and traditional mindfulness. Further analyses were then conducted on delivery methods specific to each approach (Appendix 6).

For online mindfulness interventions, all studies have reported the duration of the intervention implementation as well as the technical platform used. Based on the previous meta-analysis (Virgili, 2015; Tan et al., 2022), the classification of the duration of implementation in this study was as follows: short-term (≤ 4 weeks), medium-term (4–8 weeks), and long-term (≥ 8 weeks). The results show that the short-term (SMD = −0.97, 95% CI: −1.34, −0.59) online mindfulness intervention suggests a larger effect size than the medium-term (SMD = −0.44, 95% CI: −0.80, −0.08) and long-term (SMD = −0.34, 95% CI: −0.67, −0.02) interventions. These findings should be interpreted cautiously due to small subgroup sizes and high heterogeneity. Regarding the implementation technology platforms for online mindfulness interventions, the effects of the three technologies - App, audio, and books - were compared. The order of effectiveness from best to worst is audio (SMD = −0.65, 95% CI: −0.93, −0.37), book (SMD = −0.50, 95% CI: −0.10, −0.00), and App (SMD = −0.44, 95% CI: −0.80, −0.08). In addition, five studies reported intervention implementation frequency, which was divided into two types: daily and weekly. The intervention strategy implemented daily (SMD = −0.62, 95% CI: −0.92, −0.32) is more effective than weekly (SMD = −0.51, 95% CI: −0.84, −0.18).

For traditional mindfulness interventions, all eight studies reported the duration of the intervention implementation. Among them, the medium-term (SMD = −0.78, 95% CI: −1.12, −0.45) intervention had the best effect, which was significantly better than the short-term (SMD = −0.51, 95% CI: −0.81, −0.22) effect, while the long-term (SMD = −0.67, 95% CI: −1.02, −0.32) intervention’s effect was between the two. Apart from one study that involved a 5-day intensive training, the remaining 7 studies all maintained the intervention frequency of once per week, but the implementation times varied. The implementation time is divided into intervals of each hour: less than or equal to 1 h, 1–2 h, and greater than or equal to 2 h. The results show that as the duration of study per week increases, the effectiveness of the mindfulness intervention becomes increasingly poor. Among them, the ≤1 h group showed SMD = −1.06 (95% CI: −1.65, −0.47), the 1–2 h group showed SMD = −0.65 (95% CI: −1.05, −0.25), and the ≥2 h group showed SMD = −0.61 (95% CI: −0.85, −0.37). Moreover, six studies reported on adherence mechanisms. Among these, three utilized self - monitoring approaches (e.g., log recording), while three employed group - monitoring (e.g., experience-sharing within groups). Compared with group - monitoring (SMD = −0.78, 95% CI: −1.12, −0.37), the effect of mindfulness intervention under self - monitoring (SMD = −0.54, 95% CI: −0.81, −0.28) is more obvious.

### 4.2 Mindfulness outcome (secondary)

#### 4.2.1 Overall effects of mindfulness

**Figure 5:**
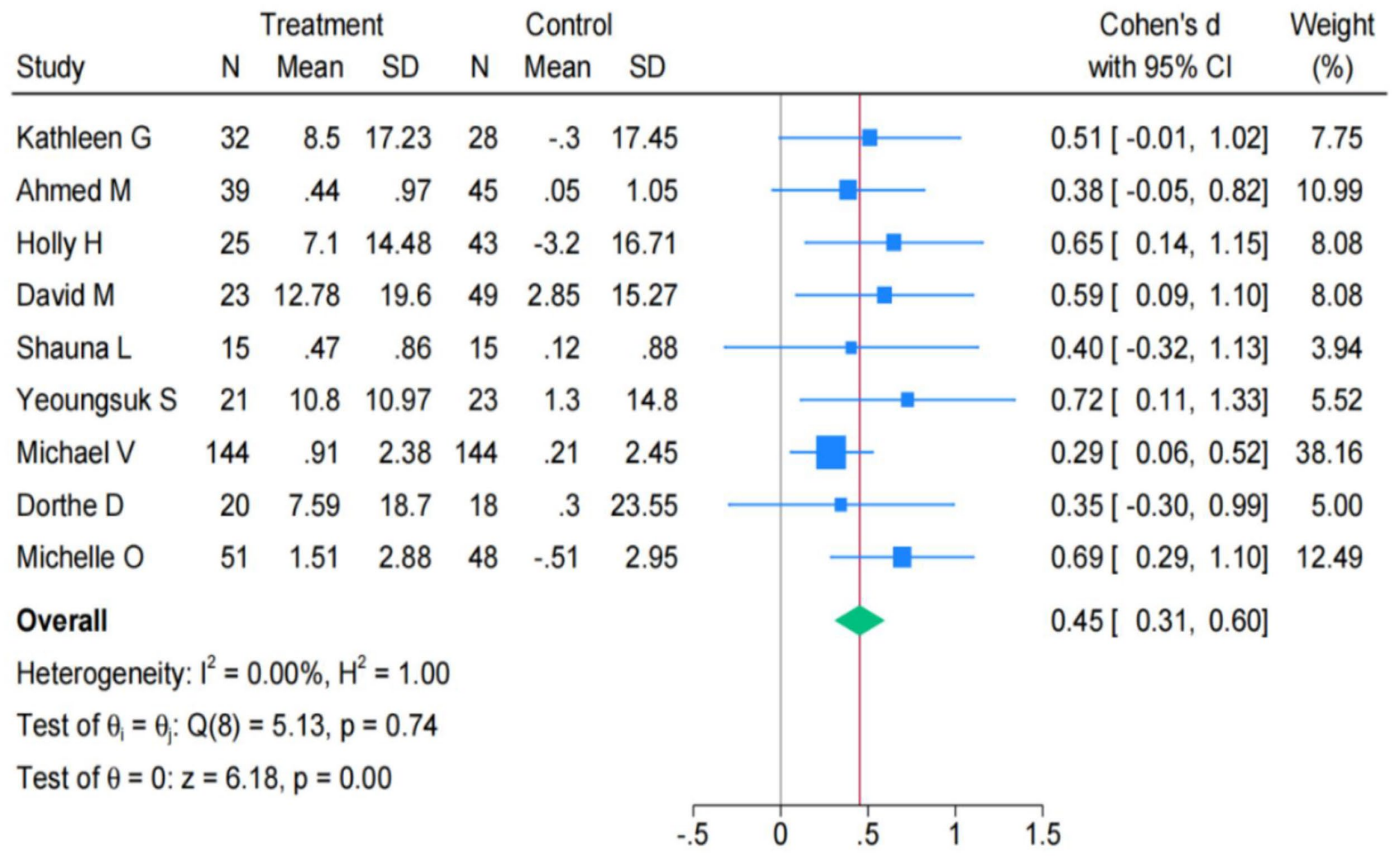
Forest plot for mindfulness (*k* = 9).

Among the included studies, 11studies evaluated changes in the level of mindfulness. However, Yang et al. (2018) only reported the observed dimensions of the FFMQ scale that were used. Therefore, a total of 10 studies were included in the final analysis. The study found significant heterogeneity after examining effects of online and traditional mindfulness interventions on mindfulness [Q(9) = 59.75, *p* < 0.001; I^2^ = 84.94%] (Appendix 7). The Galbraith plot showed a significant degree of heterogeneity in the F1 statistic (Appendix 8). After eliminating these studies, heterogeneity decreased to a small effect size [Q(8) = 5.13, *p* = 0.74; I^2^ = 0.00%] (Figure 5). Whether it is online or traditional mindfulness intervention, it can significantly enhance the mindfulness level of university students. The pooled effect size is SMD = 0.45 (95% CI: 0.31 to 0.60), and the intervention effect has a highly significant statistical meaning (z = 6.18, *p* < 0.001).

#### 4.2.2 Effects of mindfulness in subgroup

**Figure 6:**
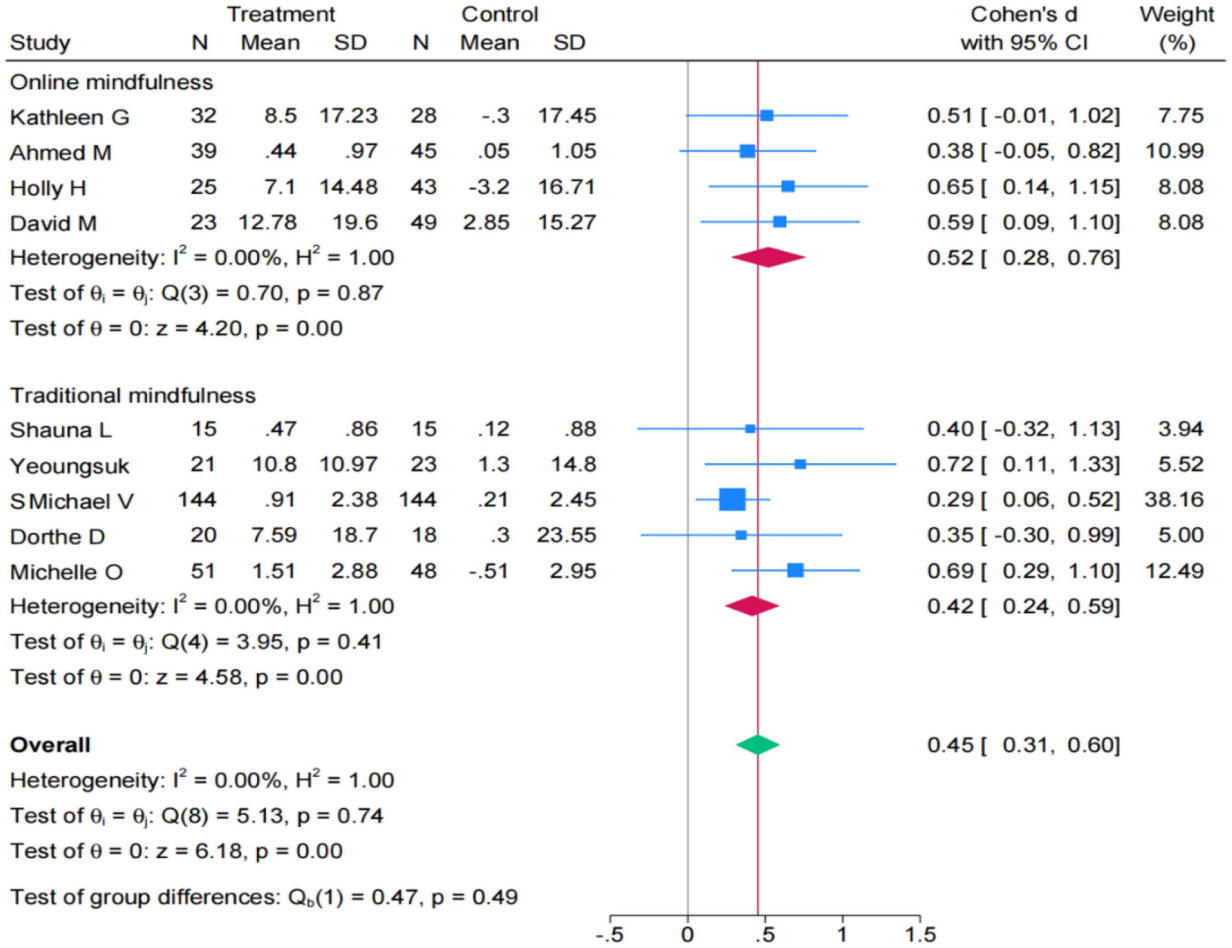
Forest plot of subgroup for mindfulness (*k* = 9).

A further subgroup analysis results indicate that both online mindfulness interventions SMD = 0.52 (95% CI: 0.28 to 0.76) and traditional mindfulness interventions SMD = 0.42 (95% CI: 0.24 to 0.59) effectively improving mindfulness, with comparable effect sizes. Studies have shown that both of these intervention models have certain effects in enhancing the mindfulness of university students. Furthermore, there was no statistically significant difference in the effect size between the two intervention methods (*p* = 0.49), indicating that the effects of these two intervention approaches are comparable (Figure 6).

#### 4.2.3 Effects of mindfulness in student subgroups

The study compared mindfulness levels between general university students and healthcare students (Table 3). For the general student population, the pooled effect size of online mindfulness intervention is SMD = 0.62 (95% CI: 0.26 to 0.98), while the pooled effect size of traditional mindfulness intervention is SMD = 0.37 (95% CI: −0.11 to 0.85). The results show that for these students, online mindfulness intervention has a medium effect size on mindfulness levels, while traditional mindfulness intervention has a smaller effect size. However, there is no significant difference between the two interventions (*p* = 0.41). For healthcare students, the pooled effect size of online mindfulness intervention is SMD = 0.44 (95% CI: 0.10 to 0.77), while the pooled effect size of traditional mindfulness intervention is SMD = 1.23 (95% CI: 0.95 to 1.52). The research results indicate that for medical students, traditional mindfulness intervention is more effective in enhancing mindfulness, and there is a statistically significant difference between the two (*p* = 0.00) (Appendix 9).

**Table 3:** Summary of intervention effects on stress in delivery-mode subgroups.

Approach	Delivery method		Studies (*N*)	Sample size	SMD [95% CI]	Two-tailed test	
						Z	*P*
Online mindfulness	Duration	4 weeks	2	132	−0.97 [−1.34, −0.59]	−5.07	0
		4–8 weeks	2	125	−0.44 [−0.80, −0.08]	−2.39	0.02
		≥ 8 weeks	2	152	−0.34 [−0.67, −0.02]	−2.07	0.04
	Technology Platform	App	2	125	−0.44 [−0.80, −0.08]	−2.39	0.02
		Audio	3	216	−0.65 [−0.93, −0.37]	−4.53	0
		Book	1	68	−0.50 [−0.10, −0.00]	−1.95	0.05
	Frequency	Daily	3	185	−0.62 [−0.92, −0.32]	−4.07	0
		Weekly	2	156	−0.51 [−0.84, −0.18]	−3.01	0
Traditional mindfulness	Duration	4 weeks	3	188	−0.51 [−0.81, −0.22]	−3.43	0
		4–8 weeks	2	150	−0.78 [−1.12, −0.45]	−4.62	0
		≥ 8 weeks	3	131	−0.67 [−1.02, −0.32]	−3.73	0
	Duration of study per week	1 h	1	51	−1.06 [−1.65, −0.47]	−3.51	0
		1–2 h	2	103	−0.65 [−1.05, −0.25]	−3.21	0
		≥ 2 h	4	277	−0.61 [−0.85, −0.37]	−4.93	0
	Adherence Mechanism	Self-monitoring	3	154	−0.78 [−1.11, −0.45]	−4.64	0
		Group-monitoring	3	233	−0.54 [−0.81, −0.28]	−4.06	0

#### 4.2.4 Effects of mindfulness in delivery-mode subgroups

Since this meta-analysis focused on stress reduction in university students, only 9 of the included studies reported mindfulness effects. Of these, four evaluated online mindfulness interventions, and 5 assessed offline interventions. Thus, subgroup analysis of intervention Delivery-Mode had limited statistical power due to scarce data (Appendix 10).

Regarding online mindfulness, a total of 4 studies were finally included. All presented the duration of the intervention as well as the technical platforms involved. In this analysis, the duration of online mindfulness interventions was categorized into short-term and long-term groups. Comparative analysis revealed no significant difference in intervention effects between these two duration groups, with the short-term group showed SMD = 0.55 (95% CI: 0.19 to 0.91) and the long-term group showed SMD = 0.50 (95% CI: 0.17 to 0.82). The technical platform only covers two types (audio and books). Compared with audio (SMD = 0.48, 95% CI: 0.21 to 0.76), the mindfulness effect of book (SMD = 0.65, 95% CI: 0.14 to 1.15) is slightly better. Furthermore, only three reported the implementation frequency in these four studies. Implementation frequency showed little effect variation between daily (SMD = 0.47, 95% CI: 0.14 to 0.80) and weekly schedules. (SMD = 0.51, 95% CI: −0.01 to 1.02).

For traditional mindfulness interventions, among the 5 studies that conducted subgroup analyses: one study reported neither weekly duration nor supervision mechanisms; one study had a weekly duration ≥2 h but did not report supervision mechanisms; the remaining three studies had weekly durations ≥2 h and used self-monitoring mechanisms. Therefore, the study can only analyze the impact of the duration of the intervention on the effect of mindfulness. The results show that the medium-term (SMD = 1.56, 95% CI: −0.15 to 3.27) traditional mindfulness intervention has a significantly better effect than the short-term (SMD = 0.35, 95% CI: −0.30 to 0.99) and long-term (SMD = 0.59, 95% CI: 0.12 to 1.06) interventions.

### 4.3 Sensitivity analysis

The pooled effect size is obtained from the stress outcome analysis was SMD = −0.6. After conducting a sensitivity analysis on this outcome, the data results showed that when all the studies were excluded one by one, the combined Cohen’s d value of the remaining studies did not change significantly (*p* < 0.05). The pooled effect size is obtained from the mindfulness outcome analysis was SMD = 0.45. A same sensitivity analysis is conducted on this result. Therefore, the results of both the meta-analysis on the outcome of stress and the meta-analysis on the outcome of mindfulness are stable and reliable. The results are shown in the Appendix 11.

A sensitivity analysis excluding the seven studies rated as high risk of bias was performed for the primary stress outcome. The recalculated pooled effect size was SMD = −0.55 (95% CI: −0.72 to −0.39), which is consistent in magnitude and direction with the primary analysis including all studies (SMD = −0.58). This suggests that the overall conclusion is not substantially altered by the inclusion of higher-risk studies, though the precision of the estimate is reduced (Table 4).

**Table 4:** Summary of effects of mindfulness intervention in student subgroup.

Variable	Subgroup	Studies (*N*)	Sample size	SMD [95% CI]	Two-tailed test	
					Z	*P*
General students	Online mindfulness	2	140	0.62 [0.26, 0.98]	3.4	0
	Traditional mindfulness	2	68	0.37 [−0.11, 0.85]	1.51	0.13
Healthcare students	Online mindfulness	2	144	0.44 [0.10, 0.77]	2.58	0.01
	Traditional mindfulness	3	247	1.24 [0.96, 1.52]	8.61	0

### 4.4 Publication bias

According to the results of the funnel plots, both the funnel plots for the stress outcomes and the mindfulness outcomes show overall symmetry with no significant gaps. Among them, the symmetry of the stress outcomes is particularly evident. However, in order to avoid subjective influence, an Egger test will be further conducted. Among them, the Egger test result for the stress outcome was z = −1.52, *p* = 0.129, and the Egger test result for the mindfulness outcome was z = 1.34, *p* = 0.181. The above test results fully confirm that, whether it is the outcome of stress or the outcome of mindfulness, there is no significant publication bias. The results are shown in the Appendix 12.

## 5 Discussion

This meta-analysis was conducted to examine the differences in the impact of online mindfulness and traditional mindfulness on the stress levels of university students. The analysis of 14 studies indicates that both online mindfulness and traditional mindfulness can effectively reduce the stress of university students. Moreover, the intervention effects of the two are quite similar. Furthermore, online and traditional mindfulness demonstrate remarkably similar intervention effects. As a secondary outcome, this study analyzed positive effects of both interventions. Results indicated that both online and traditional mindfulness significantly improved university students’ mindfulness to a comparable degree.

### 5.1 The impact of online and traditional mindfulness on university students’ stress

It is important to acknowledge that traditional mindfulness interventions often benefit from the therapeutic alliance and group support inherent in face-to-face settings. Online formats, while offering convenience and accessibility, may lack this immediate interpersonal component. However, many digital interventions incorporate elements designed to mitigate this, such as guided audio/video from experienced instructors, asynchronous discussion forums, periodic check-in messages, or integration with brief tele-coaching. Future development of online mindfulness platforms could enhance relational support through blended models (e.g., combining self-guided app use with optional live virtual sessions) to better address the need for human connection while retaining the scalability and accessibility of digital delivery.

Regarding the stress reduction situation of university students, the effect of traditional mindfulness intervention is moderate, and this result is consistent with those of previous studies (Pan et al., 2024), and the results of the online mindfulness intervention also showed a moderate effect (Sommers-Spijkerman et al., 2021; Gong et al., 2023).

Further analysis of different student groups revealed that both online mindfulness and traditional mindfulness had moderate to high levels of effectiveness for both general students and healthcare students. It is worth noting that while traditional mindfulness interventions are generally slightly more effective than online ones, the difference is not significant (Sperling et al., 2023). From this, it can be seen that for any type of university student, online mindfulness can serve as a supplement or even a replacement for offline mindfulness, this aligns with the study of Sommers-Spijkerman et al. (2021).

Based on the further analysis of the delivery mode on the stress-reducing effect of online mindfulness therapy, the following conclusion is drawn. In terms of intervention duration, the short-term mindfulness intervention had a significant effect in reducing the stress of university students. As the duration of the intervention increased, the effect gradually decreased. Furthermore, the differences in the stress-reducing effects among the three duration-based groups were statistically significant (*p* = 0.03). A small-scale study based on the “Headspace” application compared the effects on the participants during the intervention period (on the 10th day) and at the end of the intervention (on the 30th day). The results showed that the intervention effect was better on the 10th day (Champion et al., 2018). This is different from the results of Tan et al. (2022) meta-analysis, where he believed that an intervention duration of 8 weeks or more would yield better results. However, as Ana Howarth et al. (2019) pointed out, the definition of a brief MBI will affect how researchers distinguish subgroups based on the duration of the intervention. Therefore, scholars will need to systematically define and differentiate the implementation periods of mindfulness intervention in the future.

The analysis reveals that the intervention effect is superior with daily practice compared to a weekly schedule. In this study, although the total weekly practice time was similar, the distributed practice of short (10–20 min) daily sessions appears to be more effective than the massed practice of a single 2.5-h weekly session. This may be because short-term mindfulness practice is easier to maintain and involves less stress (Strohmaier et al., 2021; Sperling et al., 2023), especially for individuals who have no prior experience with mindfulness (Bonamo et al., 2015; Strohmaier et al., 2021). This is also reflected in the subgroup analysis of traditional mindfulness, which showed a statistically significant superior effect for practices lasting ≤1 h per session compared to longer sessions.

And given that online mindfulness is an approach that facilitates brief practice, we should thus prioritize exploring the technical platforms for its implementation. The studies included in this research cover audio and application software, as well as a platform for implementing intervention through reading books, because nowadays books come in the form of e-books. The results show that the online mindfulness intervention through audio delivery has a slightly better effect than the interventions on the other two types of techniques. Adherence is another critical factor influencing the effectiveness of online mindfulness. The included literature in this study measured adherence through various methods such as self-reflection (through diaries), answering questions, and clicking links. We were unable to conduct an analysis due to the limited number of studies. However, the effectiveness of the adherence approach varies in traditional mindfulness, with self-monitoring yielding better results compared to group monitoring.

Although online and traditional mindfulness are equally effective, the former offers advantages in convenience, privacy, and cost. Therefore, it can serve as a practical tool for university students to maintain their emotional wellbeing on a daily basis (Pan et al., 2024). When developing application software, it is necessary to remind the developers to consider the diversity of information dissemination (such as audio, video, e-books, and tweets). Furthermore, learning exercises should be kept short to maintain engagement, and self-monitoring tools—such as a practice log calendar and reminders—should be provided. Simultaneously, incentive strategies must be implemented to increase user engagement (Mani et al., 2015). It is also essential to evaluate the need for supervisory guidance or to implement additional support systems for questions (Sommers-Spijkerman et al., 2021).

### 5.2 The impact of online and traditional mindfulness on university students’ mindfulness

The outcomes of our study are consistent with the results of several previous studies (Querstret et al., 2020; da Silva et al., 2023; González-Martín et al., 2023). We found that both online and traditional mindfulness intervention methods resulted in a certain degree of improvement in university students’ mindfulness levels, although the overall effects were less pronounced than those observed in stress reduction.

In the subgroup analysis by student category, there were some notable findings. We found that for general students, online mindfulness intervention was more effective at increasing mindfulness levels than the traditional format. In contrast, traditional mindfulness intervention was significantly more effective for medical students; however, this finding exhibited considerable heterogeneity (I^2^ = 93.6%), suggesting a need for cautious interpretation. This heterogeneity was also observed in previous studies (Sommers-Spijkerman et al., 2021; da Silva et al., 2023), where it was at a moderate level (46–71%). We did not further analyze the source of its heterogeneity; however, based on the existing literature, there is evidence to confirm that they generally experience higher levels of stress than ordinary students (Hathaisaard et al., 2022; Sperling et al., 2023; Kaisti et al., 2024). The sources of their stress include not only the complexity of their courses but also the challenges of dealing with illness and personal life. Therefore, for medical students, in the traditional mindfulness environment, they can enhance their social support through interactions with peers and mentors (Kaisti et al., 2024), creating a safe and inclusive environment and thereby strengthening their self-awareness.

In the analysis of various delivery methods of online mindfulness, the results showed that the short-term, weekly implementation approach and the book format were numerically more effective. However, due to the lack of data for some subgroups (such as the duration of the mid-term period and the delivery of the app), a comprehensive direct comparison cannot be conducted. Therefore, it is difficult to draw precise conclusions regarding the relative effectiveness of different delivery methods. Furthermore, none of the inter-group differences reached statistical significance. This is likely due to the limited number of studies within each subgroup, resulting in insufficient statistical test power. It is worth noting that in the analysis of the duration of traditional mindfulness intervention, the mid-term intervention showed a significant effect size (SMD = 1.56). However, this result was accompanied by extremely high heterogeneity (I^2^ = 96.4%), indicating that this effect value was not stable and was strongly influenced by individual extreme studies or potential confounding factors.

Based on these findings, we suggest that future research should provide as detailed a description as possible of the implementation details of the intervention (such as the frequency of the intervention, the duration of each session, and the follow-up period), in order to clearly determine under what specific conditions the mindfulness intervention can achieve the best results.

### 5.3 Strengths and limitations

This study constitutes the first meta-analysis dedicated exclusively to Mindfulness-Based Stress Reduction (MBSR) techniques, while also being the first to systematically compare the stress reduction efficacy between online and traditional mindfulness interventions among university students. Furthermore, using the included studies, the research performed subgroup analyses on key implementation variables (including intervention duration in weeks, session frequency, and single-session duration), uncovering the dose–response relationship between intervention intensity and stress-reduction outcomes.

However, several limitations were identified during this study. Firstly, the number of included studies was insufficient. This limitation is particularly evident in the meta-analysis that assesses the subgroups of the effects of online and traditional mindfulness interventions. Furthermore, we also conducted a statistic covering aspects such as whether the participants had mindfulness experience, the qualifications of the instructors, and the long-term intervention effects. However, due to the insufficient quantity, subgroup analysis could not be conducted. Secondly, although we conducted a series of subgroup analyses, the results of some subgroups, such as the traditional mindfulness intervention group for medical students and the mid-term assessment group that received traditional mindfulness intervention, still showed significant heterogeneity. Since we did not explore the sources of this heterogeneity, the results should be interpreted with caution. Fourly, only randomized controlled trials (RCTs) with control groups (or waitlist control groups) were included. Although the research results indicate a relatively low level of heterogeneity, the exclusion of direct comparative studies between online and traditional mindfulness, as well as high-quality single-arm studies, may have an impact on the reliability of the research results. Fifth, our search was limited to English-language publications. Relevant studies published in other languages may have been missed, which could introduce language bias and limit the generalizability of findings to non-Anglophone educational contexts. Furthermore, we did not systematically search grey literature or trial registries. While our funnel plot and Egger’s test did not indicate significant publication bias for our primary outcome, this remains a potential limitation, as unpublished studies or those with null results may have been omitted, potentially inflating the overall effect size estimate. The overall certainty of the evidence is moderated by the risk of bias in the included trials. Only one study was rated as low risk, with the majority having ‘some concerns’ or ‘high risk,’ primarily due to issues with blinding and missing outcome data. While our sensitivity analysis excluding high-risk studies yielded a similar effect estimate, the prevalence of these methodological limitations necessitates cautious interpretation of the pooled effects and suggests that higher-quality, well-reported RCTs are needed in this field. Second, the generalizability of our findings is constrained by the characteristics of the included study samples. The majority of participants were female, and a significant portion were healthcare students (e.g., medical, nursing), who may experience unique academic stressors. Furthermore, most trials were conducted in Western, high-income educational contexts. Consequently, the applicability of these results to more gender-balanced populations, students from non-healthcare disciplines, and diverse cultural or educational settings (e.g., in low- and middle-income countries) requires further investigation.

Additionally, a growing body of research highlights the stress-reducing benefits of contact with nature. None of the included online mindfulness interventions explicitly integrated nature-based elements (e.g., guided practice in natural settings, nature visuals/sounds). Future digital interventions could explore hybrid approaches that combine mindfulness training with ecotherapy principles or nature exposure, potentially enhancing their effectiveness for stress reduction.

## 6 Conclusion

In conclusion, this meta-analysis indicates that online mindfulness-based interventions appear to be a viable and similarly effective alternative to traditional formats for reducing stress in university students, based on indirect comparisons with control conditions. While online formats may lack the interpersonal immediacy of face-to-face groups, they offer distinct advantages in accessibility, scalability, cost-effectiveness, and privacy. These features make them particularly suitable for reaching students in remote locations, those with scheduling constraints, or individuals who prefer self-paced, private engagement. For universities with limited mental health resources, online mindfulness can serve as a scalable first-line or adjunctive support. Future iterations should consider incorporating elements of social support, personalization, and even nature-based content to enhance engagement and outcomes. Thus, online mindfulness represents not necessarily a replacement, but a complementary and highly adaptable tool within a comprehensive university mental health strategy.

## Data Availability

The original contributions presented in the study are included in the article/Supplementary material, further inquiries can be directed to the corresponding authors.

## References

[ref1] Alhmoud J. F. Farah H. S. Al-Qaisi T. (2021). The changes in some hematological parameters among university students due to stressful conditions during and after examinations period. Indian J. Forensic Med. Toxicol. 15, 1181–1186. doi: 10.37506/ijfmt.v15i1.13578

[ref9001] Alrashdi D. H. Chen K. K. Meyer C. Gould R. L. (2024). A systematic review and meta-analysis of online mindfulness-based interventions for university students: an examination of psychological distress and well-being, and attrition rates. Journal of Technology in Behavioral Science, 9, 211–223. doi: 10.1007/s41347-023-00321-6

[ref2] Alsaqer K. Al-Maghaireh D. Kawafha M. Sheyab H. Al Kofahi A. Saleh M. (2025). The effects of mindfulness-based stress reduction on depression, anxiety, stress, and mindfulness among undergraduate nursing students in the psychiatric clinical practice. Mindfulness 16, 1250–1257. doi: 10.1007/s12671-025-02559-y

[ref3] Alzahrani A. M. Hakami A. AlHadi A. Al-Maflehi N. Aljawadi M. H. Alotaibi R. M. . (2023). The effectiveness of mindfulness training in improving medical students’ stress, depression, and anxiety. PLoS One 18:e0293539. doi: 10.1371/journal.pone.0293539, 37906599 PMC10617730

[ref4] Andersson C. Bergsten K. L. Lilliengren P. Norbäck K. Rask K. Einhorn S. . (2021). The effectiveness of smartphone compassion training on stress among Swedish university students: a pilot randomized trial. J. Clin. Psychol. 77, 927–945. doi: 10.1002/jclp.23092, 33245161

[ref5] Baminiwatta A. Solangaarachchi I. (2021). Trends and developments in mindfulness research over 55 years: a bibliometric analysis of publications indexed in web of science. Mindfulness 12, 2099–2116. doi: 10.1007/s12671-021-01681-x, 34306245 PMC8282773

[ref6] Beblo T. Haehnel K. Michalak J. Iffland B. Driessen M. (2024). Integrating mindfulness practice into everyday life after completing a course in mindfulness-based stress reduction. Nord. Psychol. 76, 506–518. doi: 10.1080/19012276.2024.2303432

[ref7] Belmans E. Bastin M. Raes F. Bijttebier P. (2019). Temporal associations between social anxiety and depressive symptoms and the role of interpersonal stress in adolescents. Depress. Anxiety 36, 960–967. doi: 10.1002/da.22939, 31437332

[ref8] Böke B. N. Mills D. J. Mettler J. Heath N. L. (2019). Stress and coping patterns of university students. J. Coll. Stud. Dev. 60, 85–103. doi: 10.1353/csd.2019.0005

[ref9] Bonamo K. K. Legerski J. P. Thomas K. B. (2015). The influence of a brief mindfulness exercise on encoding of novel words in female college students. Mindfulness 6, 535–544. doi: 10.1007/s12671-014-0285-3

[ref10] Borenstein M. Hedges L. V. Higgins J. P. Rothstein H. R. (2021). Introduction to meta-analysis. New York, NY: John Wiley & Sons.

[ref11] Bramer W. M. Rethlefsen M. L. Kleijnen J. Franco O. H. (2017). Optimal database combinations for literature searches in systematic reviews: a prospective exploratory study. Syst. Rev. 6:245. doi: 10.1186/s13643-017-0644-y, 29208034 PMC5718002

[ref12] Burger K. G. Lockhart J. S. (2017). Meditation's effect on attentional efficiency, stress, and mindfulness characteristics of nursing students. J. Nurs. Educ. 56, 430–434. doi: 10.3928/01484834-20170619-08, 28662260

[ref13] Champion L. Economides M. Chandler C. (2018). The efficacy of a brief app-based mindfulness intervention on psychosocial outcomes in healthy adults: a pilot randomised controlled trial. PLoS One 13:e0209482. doi: 10.1371/journal.pone.0209482, 30596696 PMC6312207

[ref14] Chandler J. Cumpston M. Li T. Page M. J. Welch V. J. H. W. (2019). Cochrane handbook for systematic reviews of interventions, vol. 4.2. Hoboken, NJ: Wiley.

[ref15] Choi J. (2020). Impact of stress levels on eating Behaviors among college students. Nutrients 12:1241. doi: 10.3390/nu12051241, 32349338 PMC7284653

[ref16] Cohen J. (2013). Statistical power analysis for the behavioral sciences. London: Routledge.

[ref17] Cohen-Katz J. Wiley S. Capuano T. Baker D. M. Deitrick L. Shapiro S. (2005b). The effects of mindfulness-based stress reduction on nurse stress and burnout: a qualitative and quantitative study, part III. Holist. Nurs. Pract. 19, 78–86. doi: 10.1097/00004650-200503000-00009, 15871591

[ref18] Cohen-Katz J. Wiley S. D. Capuano T. Baker D. M. Shapiro S. (2005a). The effects of mindfulness-based stress reduction on nurse stress and burnout, part II: a quantitative and qualitative study. Holist. Nurs. Pract. 19, 26–35. doi: 10.1097/00004650-200501000-00008, 15736727

[ref20] Dark-Freudeman A. Jones C. Terry C. (2020). Mindfulness, anxiety, and perceived stress in university students: comparing a mindfulness-based intervention (MBI) against active and traditional control conditions. J. Am. Coll. Heal. 70, 2116–2125. doi: 10.1080/07448481.2020.1845180, 33400631

[ref19] Da Silva C. C. G. Bolognani C. V. Amorim F. F. Imoto A. M. (2023). Effectiveness of training programs based on mindfulness in reducing stress and promoting wellbeing in medical students: a systematic review and meta-analysis. Syst. Rev. 12:79. doi: 10.1186/s13643-023-02244-y, 37147732 PMC10160720

[ref21] De Vibe M. Solhaug I. Tyssen R. Friborg O. Rosenvinge J. H. Sørlie T. . (2013). Mindfulness training for stress management: a randomised controlled study of medical and psychology students. BMC Med. Educ. 13:107. doi: 10.1186/1472-6920-13-107, 23941053 PMC3751423

[ref22] Djernis D. O’Toole M. S. Fjorback L. O. Svenningsen H. Mehlsen M. Y. Stigsdotter U. K. . (2021). A short mindfulness retreat for students to reduce stress and promote self-compassion: pilot randomised controlled trial exploring both an indoor and a natural outdoor retreat setting. Healthcare 9:910. doi: 10.3390/healthcare9070910, 34356288 PMC8307600

[ref23] Erogul M. Singer G. McIntyre T. Stefanov D. G. (2014). Abridged mindfulness intervention to support wellness in first-year medical students. Teach. Learn. Med. 26, 350–356. doi: 10.1080/10401334.2014.945025, 25318029

[ref24] Farris S. R. Grazzi L. Holley M. Dorsett A. Xing K. Pierce C. R. . (2021). Online mindfulness may target stress and mental health during COVID-19. Glob. Adv. Health Med. 10:21649561211002461. doi: 10.1177/21649561211002461 34497735 PMC8419565

[ref25] Firth J. Torous J. Yung A. R. (2016). Ecological momentary assessment and beyond: the rising interest in e-mental health research. J. Psychiatr. Res. 80, 3–4. doi: 10.1016/j.jpsychires.2016.05.002, 27236099

[ref26] Frank J. L. Reibel D. Broderick P. Cantrell T. Metz S. (2015). The effectiveness of mindfulness-based stress reduction on educator stress and wellbeing: results from a pilot study. Mindfulness 6, 208–216. doi: 10.1007/s12671-013-0246-2

[ref9002] Furukawa T. A. Shinohara K. Sahker E. Karyotaki E. Miguel C. Ciharova M. et al. (2021). Initial treatment choices to achieve sustained response in major depression: a systematic review and network meta‐analysis. World Psychiatry, 20, 387–396. doi: 10.1002/wps.20906 34505365 PMC8429344

[ref27] Gallup (2024). Gallup global emotions report 2024. Washington, D.C., United States: Gallup, Inc. Available online at: https://news.gallup.com/poll/645770/great-management-improve-mental-health.aspx

[ref28] Gawrysiak M. J. Grassetti S. N. Greeson J. M. Shorey R. C. Pohlig R. Baime M. J. (2018). The many facets of mindfulness and the prediction of change following mindfulness-based stress reduction (MBSR). J. Clin. Psychol. 74, 523–535. doi: 10.1002/jclp.22521, 28815600 PMC5815955

[ref29] Gong X. G. Wang L. P. Rong G. Zhang D. N. Zhang A. Y. Liu C. (2023). Effects of online mindfulness-based interventions on the mental health of university students: a systematic review and meta-analysis. Front. Psychol. 14:1073647. doi: 10.3389/fpsyg.2023.1073647, 36844353 PMC9944037

[ref30] González-Martín A. M. Aibar-Almazán A. Rivas-Campo Y. Castellote-Caballero Y. Carcelén-Fraile M. D. C. (2023). Mindfulness to improve the mental health of university students. A systematic review and meta-analysis. Front. Public Health 11:1284632. doi: 10.3389/fpubh.2023.1284632, 38111480 PMC10726040

[ref31] Hathaisaard C. Wannarit K. Pattanaseri K. (2022). Mindfulness-based interventions reducing and preventing stress and burnout in medical students: a systematic review and meta-analysis. Asian J. Psychiatr. 69:102997. doi: 10.1016/j.ajp.2021.102997, 34995839

[ref32] Hazlett-Stevens H. Oren Y. (2016). Effectiveness of mindfulness-based stress reduction bibliotherapy: a preliminary randomized controlled trial. J. Clin. Psychol. 73, 626–637. doi: 10.1002/jclp.22370, 27487300

[ref33] Higgins J. P. T. Green S. (2011). Cochrane handbook for systematic reviews of interventions (version 5.1.0). London: The Cochrane Collaboration.

[ref34] Howarth A. Smith J. G. Perkins-Porras L. Ussher M. (2019). Effects of brief mindfulness-based interventions on health-related outcomes: a systematic review. Mindfulness 10, 1957–1968. doi: 10.1007/s12671-019-01163-1

[ref35] Juruena M. F. Eror F. Cleare A. J. Young A. H. (2020). “The role of early life stress in HPA Axis and anxiety” in Anxiety disorders. Advances in experimental medicine and biology. ed. Kim Y. K. (Singapore: Springer). 10.1007/978-981-32-9705-0_9 32002927

[ref36] Kabat-Zinn J. (1982). An outpatient program in behavioral medicine for chronic pain patients based on the practice of mindfulness meditation: theoretical considerations and preliminary results. Gen. Hosp. Psychiatry 4, 33–47. doi: 10.1016/0163-8343(82)90026-3, 7042457

[ref37] Kabat-Zinn J. (1990). Full catastrophe living: Using the wisdom of the body and the mind to fase stress, pain and illness. New York, US: Dell.

[ref38] Kabat-Zinn J. (2015). Mindfulness. Mindfulness 6, 1481–1483. doi: 10.1007/s12671-015-0456-x

[ref39] Kaisti I. Kulmala P. Hintsanen M. Hurtig T. Repo S. Paunio T. . (2024). The effects of mindfulness-based interventions in medical students: a systematic review. Adv. Health Sci. Educ. 29, 245–271. doi: 10.1007/s10459-023-10231-0, 37227541 PMC10927869

[ref40] Kang H. K. Rhodes C. Rivers E. Thornton C. P. Rodney T. (2021). Prevalence of mental health disorders among undergraduate university students in the United States: a review. J. Psychosoc. Nurs. Ment. Health Serv. 59, 17–24. doi: 10.3928/02793695-20201104-03, 33180947

[ref41] Karo M. Simorangkir L. Daryanti Saragih I. Suarilah I. Tzeng H. M. (2024). Effects of mindfulness-based interventions on reducing stress among nurses: a systematic review and meta-analysis of randomized controlled trials. J. Nurs. Scholarsh. 56, 319–330. doi: 10.1111/jnu.12941, 37955233

[ref42] Larsen M. E. Nicholas J. Christensen H. (2016). Quantifying app store dynamics: longitudinal tracking of mental health apps. JMIR Mhealth Uhealth 4:e96. doi: 10.2196/mhealth.6020, 27507641 PMC4995352

[ref44] Liu Q. Liu Y. Leng X. Han J. Xia F. Chen H. (2020). Impact of chronic stress on attention control: evidence from behavioral and event-related potential analyses. Neurosci. Bull. 36, 1395–1410. doi: 10.1007/s12264-020-00549-9, 32929635 PMC7674527

[ref43] Li W. Huo S. Yin F. Wu Z. Zhang X. Wang Z. . (2024). The differences in symptom networks of depression, anxiety, and sleep in college students with different stress levels. BMC Public Health 24:3609. doi: 10.1186/s12889-024-21161-w, 39736526 PMC11684240

[ref45] Lomas T. Medina J. C. Ivtzan I. Rupprecht S. Eiroa-Orosa F. J. (2017). The impact of mindfulness on the wellbeing and performance of educators: a systematic review of the empirical literature. Teach. Teach. Educ. 61, 132–141. doi: 10.1016/j.tate.2016.10.008

[ref46] Mani M. Kavanagh D. J. Hides L. Stoyanov S. R. (2015). Review and evaluation of mindfulness-based iPhone apps. JMIR Mhealth Uhealth 3:e4328. doi: 10.2196/mhealth.4328, 26290327 PMC4705029

[ref47] Marsano L. Bardill A. Fields B. Herd K. Veale D. Grey N. . (2015). The application of mHealth to mental health: opportunities and challenges. Lancet Psychiatry 2, 942–948. doi: 10.1016/S2215-0366(15)00268-0 26462228

[ref48] Messer D. Horan J. J. Turner W. Weber W. (2015). The effects of internet-delivered mindfulness training on stress, coping, and mindfulness in university students. AERA Open 2:2332858415625188. doi: 10.1177/233285841562518

[ref9003] Moher D. Liberati A. Tetzlaff J. Altman D. G. (2009). Preferred reporting items for systematic reviews and meta-analyses: The PRISMA statement. Annals of Internal Medicine, 151, 264–269. doi: 10.1371/journal.pmed.1000097\t 19622511

[ref49] Nehra D. K. Sharma N. Kumar P. Nehra S. (2013). “Mindfulness based stress reduction (MBSR) program: an overview” in Mental health: Risks and resources. eds. Hooda D. Sharma N. R. (New Delhi: Global Vision Publishing House), 197–230.

[ref50] O’Connor D. B. Thayer J. T. Vedhara K. (2021). Stress and health: a review of psychobiological processes. Annu. Rev. Psychol. 72, 663–688. doi: 10.1146/annurev-psych-062520-122331 32886587

[ref51] O'Driscoll M. Sahm L. J. Byrne H. Lambert S. Byrne S. (2019). Impact of a mindfulness-based intervention on undergraduate pharmacy students' stress and distress: quantitative results of a mixed-methods study. Curr. Pharm. Teach. Learn. 11, 876–887. doi: 10.1016/j.cptl.2019.05.014, 31570124

[ref52] Page M. J. McKenzie J. E. Bossuyt P. M. Boutron I. Hoffmann T. C. Mulrow C. D. . (2021). The PRISMA 2020 statement: an updated guideline for reporting systematic reviews. BMJ 372:n71. doi: 10.1136/bmj.n71, 33782057 PMC8005924

[ref53] Pan Y. Li F. Liang H. Shen X. Bing Z. Cheng L. . (2024). Effectiveness of mindfulness-based stress reduction on mental health and psychological quality of life among university students: a GRADE-assessed systematic review. Evid.-Based Complement. Altern. Med. 2024:8872685. doi: 10.1155/2024/8872685, 38414520 PMC10898947

[ref54] Querstret D. Morison L. Dickinson S. Cropley M. John M. (2020). Mindfulness-based stress reduction and mindfulness-based cognitive therapy for psychological health and wellbeing in nonclinical samples: a systematic review and meta-analysis. Int. J. Stress Manage. 27:394. doi: 10.1037/str0000165

[ref55] Santorelli S. F. Kabat-Zinn J. Blacker M. Meleo-Meyer F. Koerbel L. (2017). Mindfulness-based stress reduction (MBSR) authorized curriculum guide. Center for mindfulness in medicine, health care, and society (CFM). Worcester, MA: University of Massachusetts Medical Schoold.

[ref56] Schmickler J. M. Blaschke S. Robbins R. Mess F. (2023). Determinants of sleep quality: a cross-sectional study in university students. Int. J. Environ. Res. Public Health 20:2019. doi: 10.3390/ijerph20032019, 36767422 PMC9915447

[ref57] Segal Z. Williams M. Teasdale J. (2012). Mindfulness-based cognitive therapy for depression. London: Guilford Press.

[ref58] Shapiro S. L. Brown K. W. Thoresen C. Plante T. G. (2011). The moderation of mindfulness-based stress reduction effects by trait mindfulness: results from a randomized controlled trial. J. Clin. Psychol. 67, 267–277. doi: 10.1002/jclp.20761, 21254055

[ref59] Sipe W. E. Eisendrath S. J. (2012). Mindfulness-based cognitive therapy: theory and practice. Can. J. Psychiatr. 57, 63–69. doi: 10.1177/070674371205700202, 22340145

[ref60] Smith S. A. (2014). Mindfulness-based stress reduction: an intervention to enhance the effectiveness of nurses' coping with work-related stress. Int. J. Nurs. Knowl. 25, 119–130. doi: 10.1111/2047-3095.12025, 24612607

[ref61] Sommers-Spijkerman M. Austin J. Bohlmeijer E. Pots W. (2021). New evidence in the booming field of online mindfulness: an updated meta-analysis of randomized controlled trials. JMIR Mental Health 8:e28168. doi: 10.2196/28168, 34279240 PMC8329762

[ref62] Song Y. Lindquist R. (2015). Effects of mindfulness-based stress reduction on depression, anxiety, stress and mindfulness in Korean nursing students. Nurse Educ. Today 35, 86–90. doi: 10.1016/j.nedt.2014.06.010, 25066651

[ref63] Sperling E. L. Hulett J. M. Sherwin L. B. Thompson S. Bettencourt B. A. (2023). The effect of mindfulness interventions on stress in medical students: a systematic review and meta-analysis. PLoS One 18:e0286387. doi: 10.1371/journal.pone.0286387, 37796866 PMC10553303

[ref64] Ștefan C. A. Căpraru C. Szilágyi M. (2018). Investigating effects and mechanisms of a mindfulness-based stress reduction intervention in a sample of college students at risk for social anxiety. Mindfulness 9, 1509–1521. doi: 10.1007/s12671-018-0899-y

[ref65] Strohmaier S. Jones F. W. Cane J. E. (2021). Effects of length of mindfulness practice on mindfulness, depression, anxiety, and stress: a randomized controlled experiment. Mindfulness 12, 198–214. doi: 10.1007/s12671-020-01512-5

[ref66] Sun J. (2014). Mindfulness in context: a historical discourse analysis. Contemp. Buddhism 15, 394–415. doi: 10.1080/14639947.2014.978088

[ref67] Tan Z. Y. A. Wong S. H. Cheng L. J. Lau S. T. (2022). Effectiveness of mobile-based mindfulness interventions in improving mindfulness skills and psychological outcomes for adults: a systematic review and meta-regression. Mindfulness 13, 2379–2395. doi: 10.1007/s12671-022-01962-z

[ref68] Teasdale J. D. Moore R. G. Hayhurst H. Pope M. Williams S. Segal Z. V. (2002). Metacognitive awareness and prevention of relapse in depression: empirical evidence. J. Consult. Clin. Psychol. 70, 275–287. doi: 10.1037//0022-006X.70.2.275, 11952186

[ref69] Tickell A. Ball S. Bernard P. Kuyken W. Marx R. Pack S. . (2020). The effectiveness of mindfulness-based cognitive therapy (MBCT) in real-world healthcare services. Mindfulness 11, 279–290. doi: 10.1007/s12671-018-1087-9, 32064009 PMC6995449

[ref70] Virgili M. (2015). Mindfulness-based interventions reduce stress in working adults: a meta-analysis of intervention studies. Mindfulness 6, 326–337. doi: 10.1007/s12671-013-0264-0

[ref71] Wang Y. Liao L. Lin X. Sun Y. Wang N. Wang J. . (2021). A bibliometric and visualization analysis of mindfulness and meditation research from 1900 to 2021. Int. J. Environ. Res. Public Health 18:13150. doi: 10.3390/ijerph182413150, 34948760 PMC8701075

[ref72] Wemm S. E. Wulfert E. (2017). Effects of acute stress on decision making. Appl. Psychophysiol. Biofeedback 42, 1–12. doi: 10.1007/s10484-016-9347-8, 28083720 PMC5346059

[ref73] Wheatley D. (1997). Stress, anxiety and depression. Stress Med. 13, 173–177. doi: 10.1002/(sici)1099-1700(199707)13:3<>3.0.co;2-6

[ref74] Wolever R. Q. Finn M. T. Shields D. (2022). The relative contributions of live and recorded online mindfulness training programs to lower stress in the workplace: longitudinal observational study. J. Med. Internet Res. 24:e31935. doi: 10.2196/31935, 35060911 PMC8817217

[ref75] World Health Organization (2022). World mental health report: Transforming mental health for all. Geneva: WHO.

[ref76] Wu D. Yu L. Yang T. Cottrell R. Peng S. Guo W. . (2020). The impacts of uncertainty stress on mental disorders of Chinese college students: evidence from a nationwide study. Front. Psychol. 11:243. doi: 10.3389/fpsyg.2020.00243, 32210868 PMC7075936

[ref77] Xiong J. Lipsitz O. Nasri F. Lui L. M. Gill H. Phan L. . (2020). Impact of COVID-19 pandemic on mental health in the general population: a systematic review. J. Affect. Disord. 277, 55–64. doi: 10.1016/j.jad.2020.08.001, 32799105 PMC7413844

[ref78] Yang E. Schamber E. Meyer R. M. Gold J. I. (2018). Happier healers: randomized controlled trial of mobile mindfulness for stress management. J. Altern. Complement. Med. 24, 505–513. doi: 10.1089/acm.2015.0301, 29420050

[ref79] Ying C. Liu C. He J. Wang J. (2018). Academic stress and evaluation of a mindfulness training intervention program. NeuroQuantology 16:1311. doi: 10.14704/nq.2018.16.5.1311

[ref80] Zenner C. Herrnleben-Kurz S. Walach H. (2014). Mindfulness-based interventions in schools—a systematic review and meta-analysis. Front. Psychol. 5:603. doi: 10.3389/fpsyg.2014.00603, 25071620 PMC4075476

