# Supplementary material for: Online and traditional mindfulness-based interventions for stress in university students: a systematic review and meta-analysis versus control conditions
Source: Front Psychol. 2026 Mar 27;17:1755245. doi: 10.3389/fpsyg.2026.1755245 (PMC13067905; doi:10.3389/fpsyg.2026.1755245)
Supplement: Supplementary file 1 [file Data_Sheet_1.zip › Appendix 6.pdf]

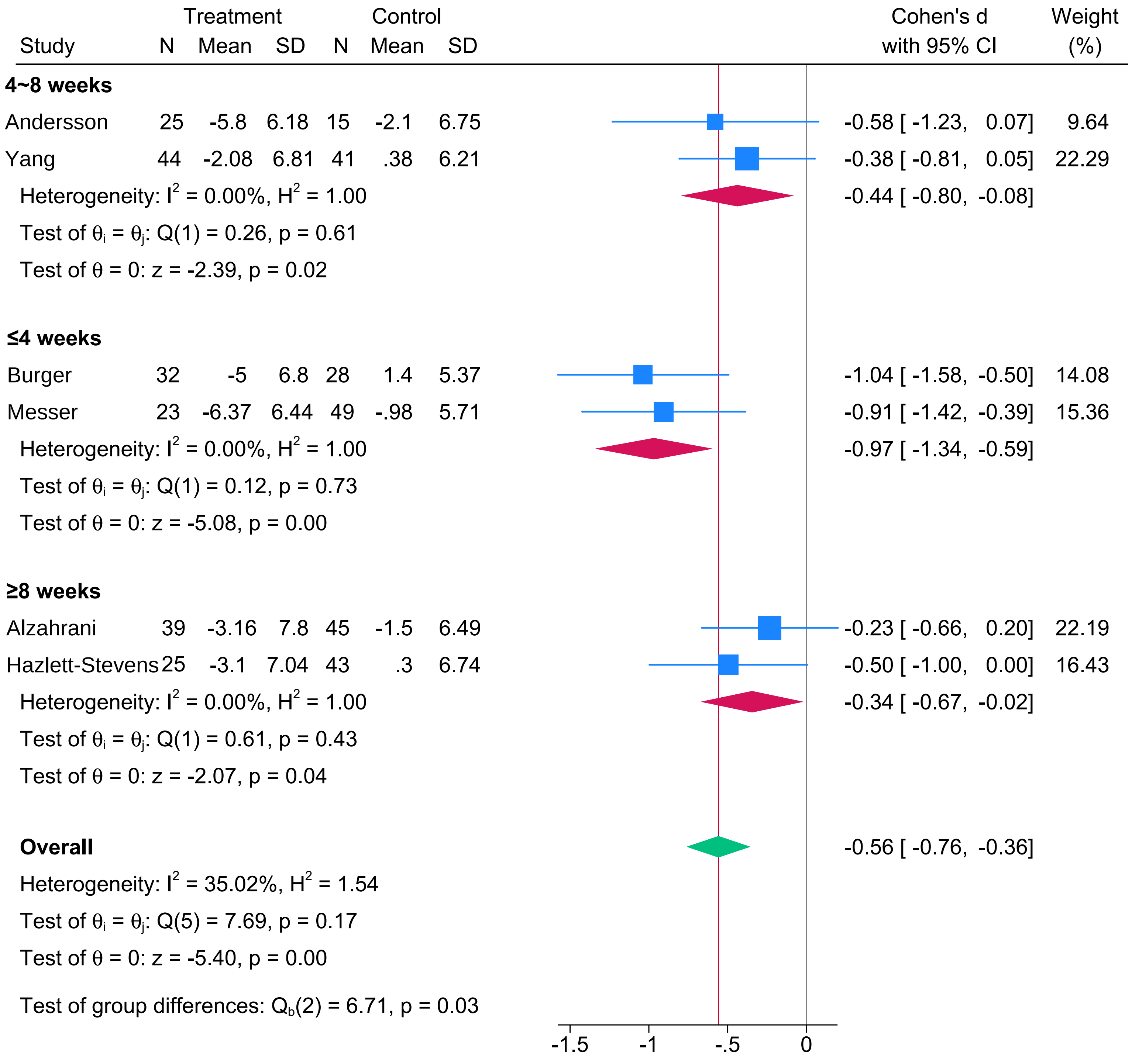

The Effect of Online Mindfulness on Stress in Duration Subgroup

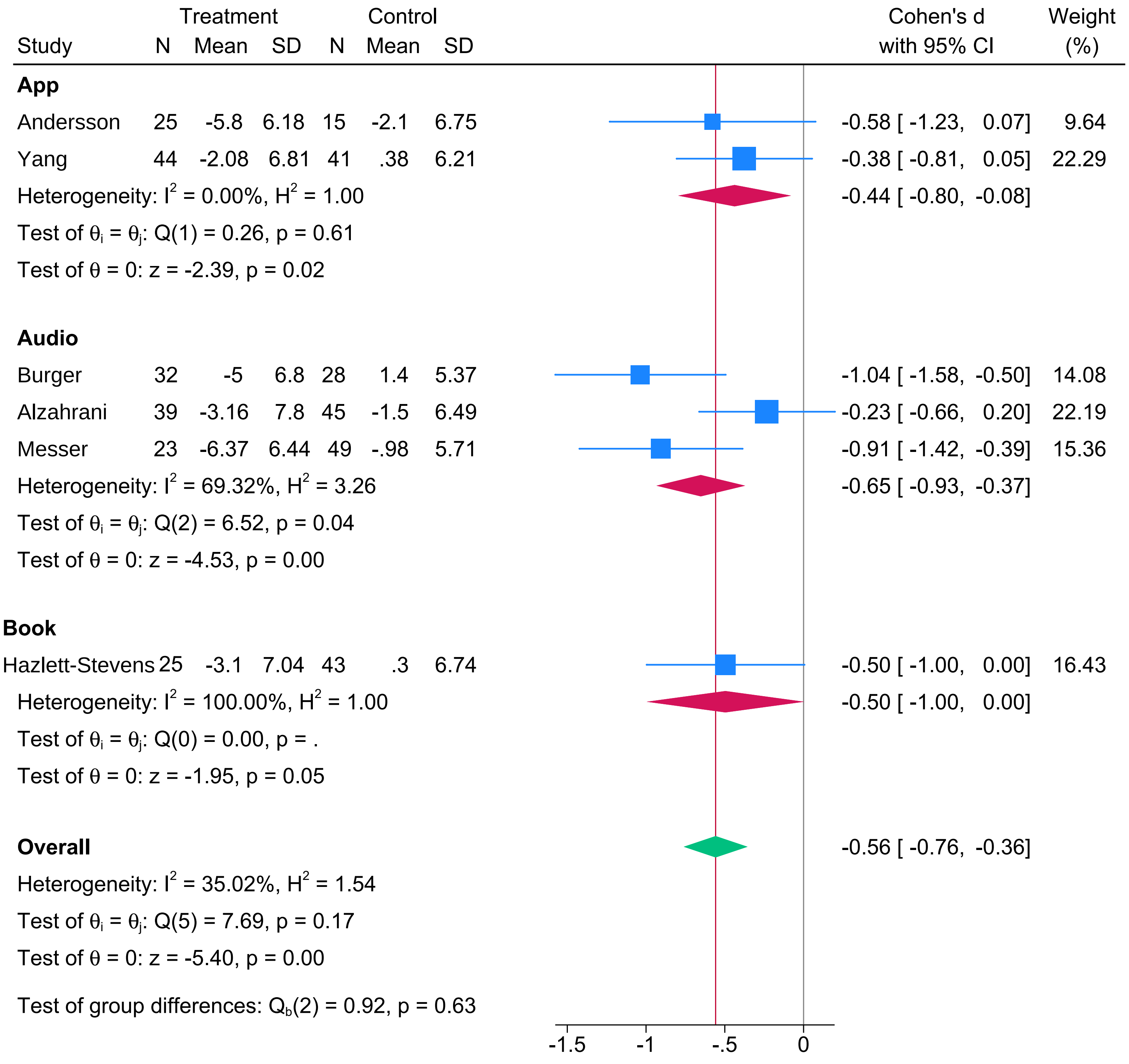

The Effect of Online Mindfulness on Stress in Technology platform Subgroup

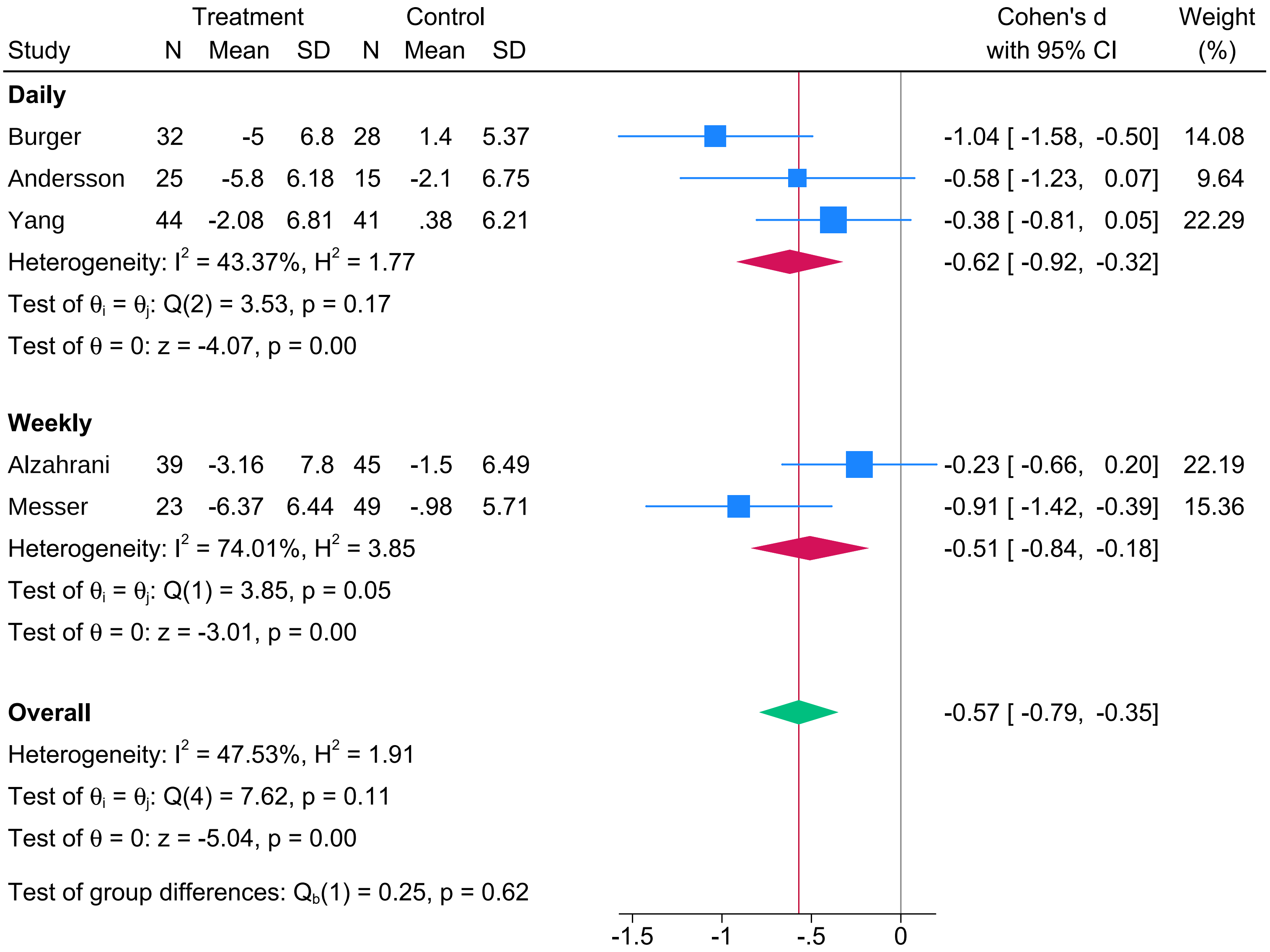

The Effect of Online Mindfulness on Stress in Frequency Subgroup

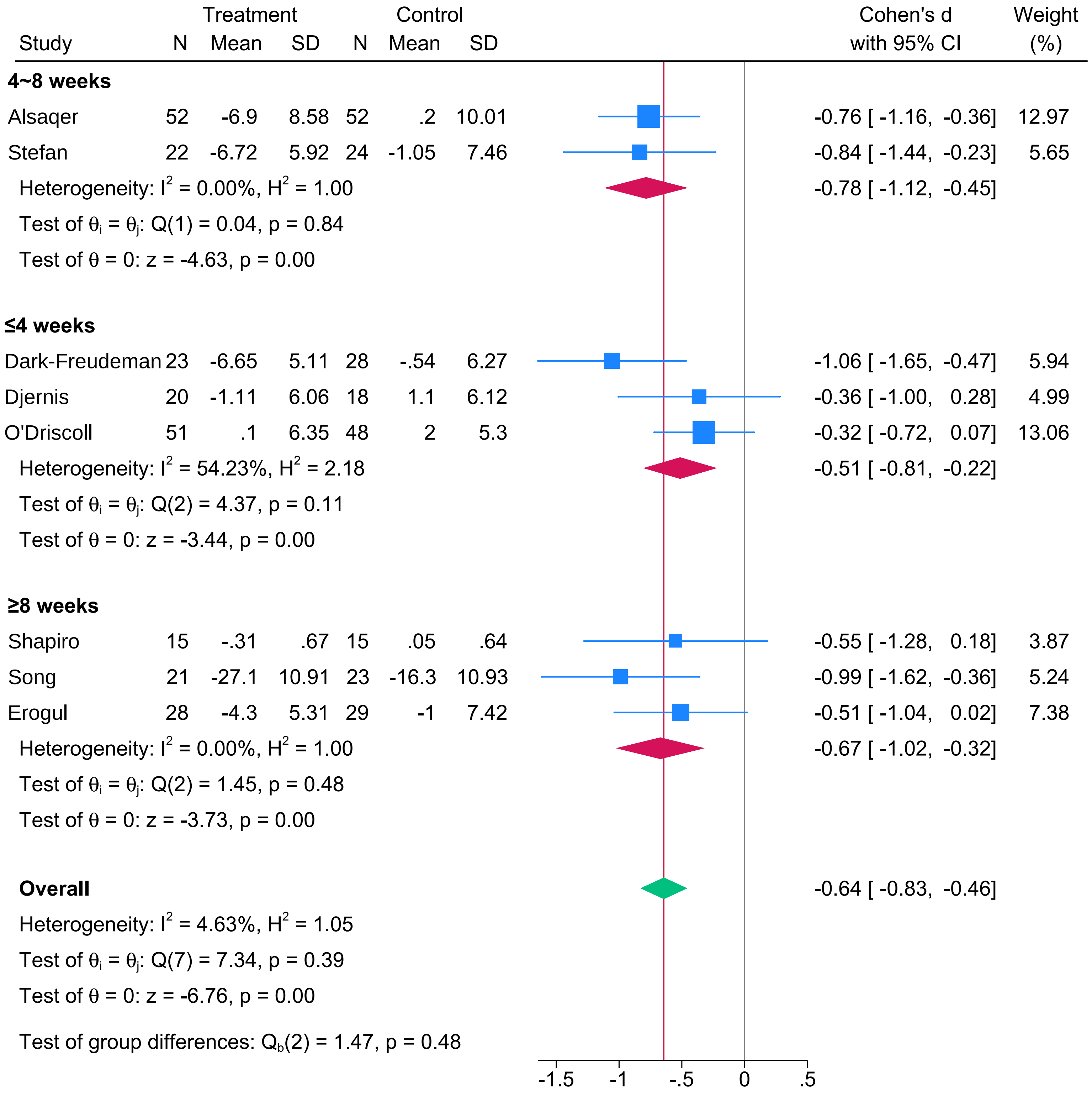

The Effect of Traditional Mindfulness on Stress in Duration Subgroup

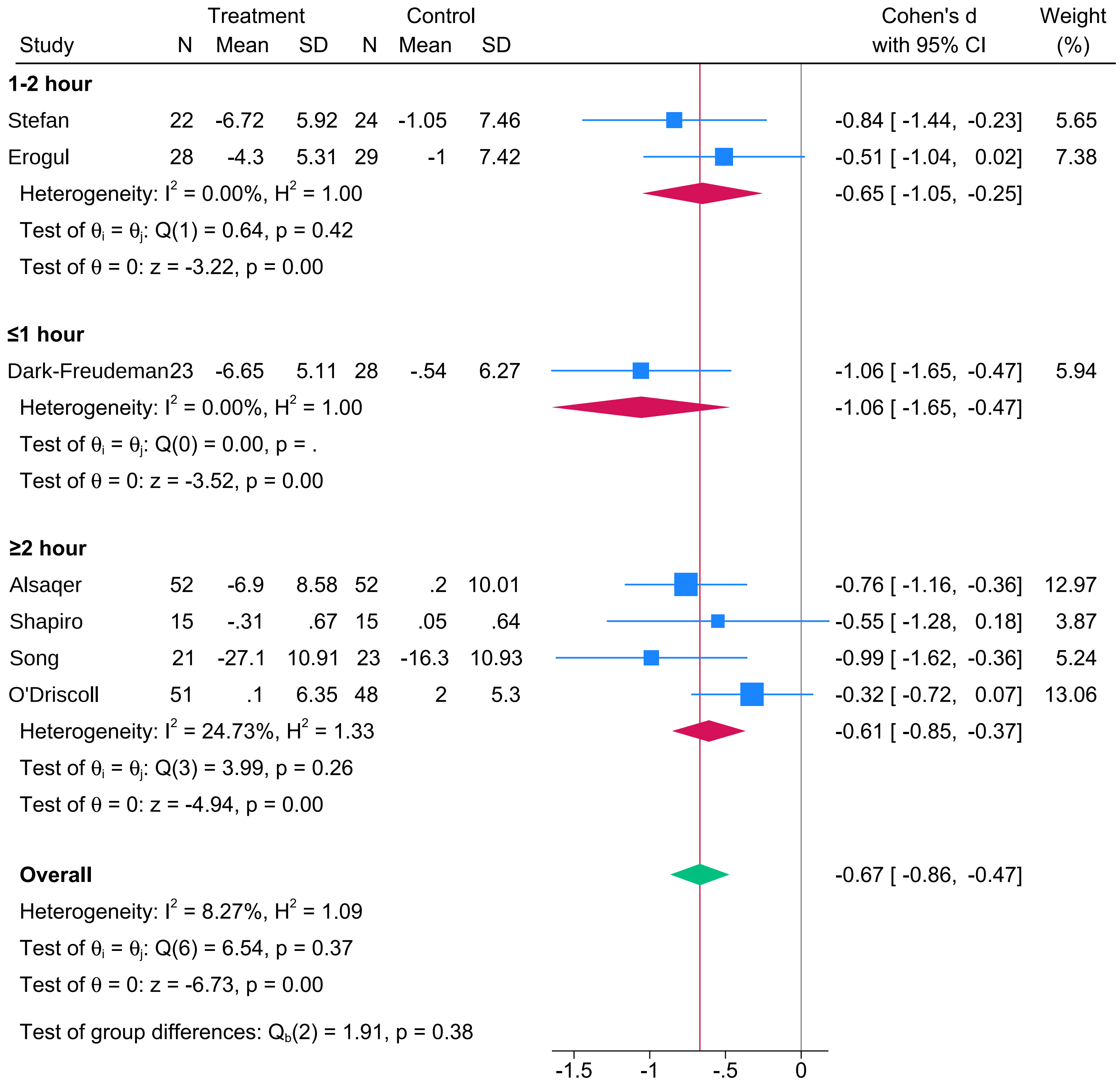

The Effect of Traditional Mindfulness on Stress in Duration of study per week Subgroup

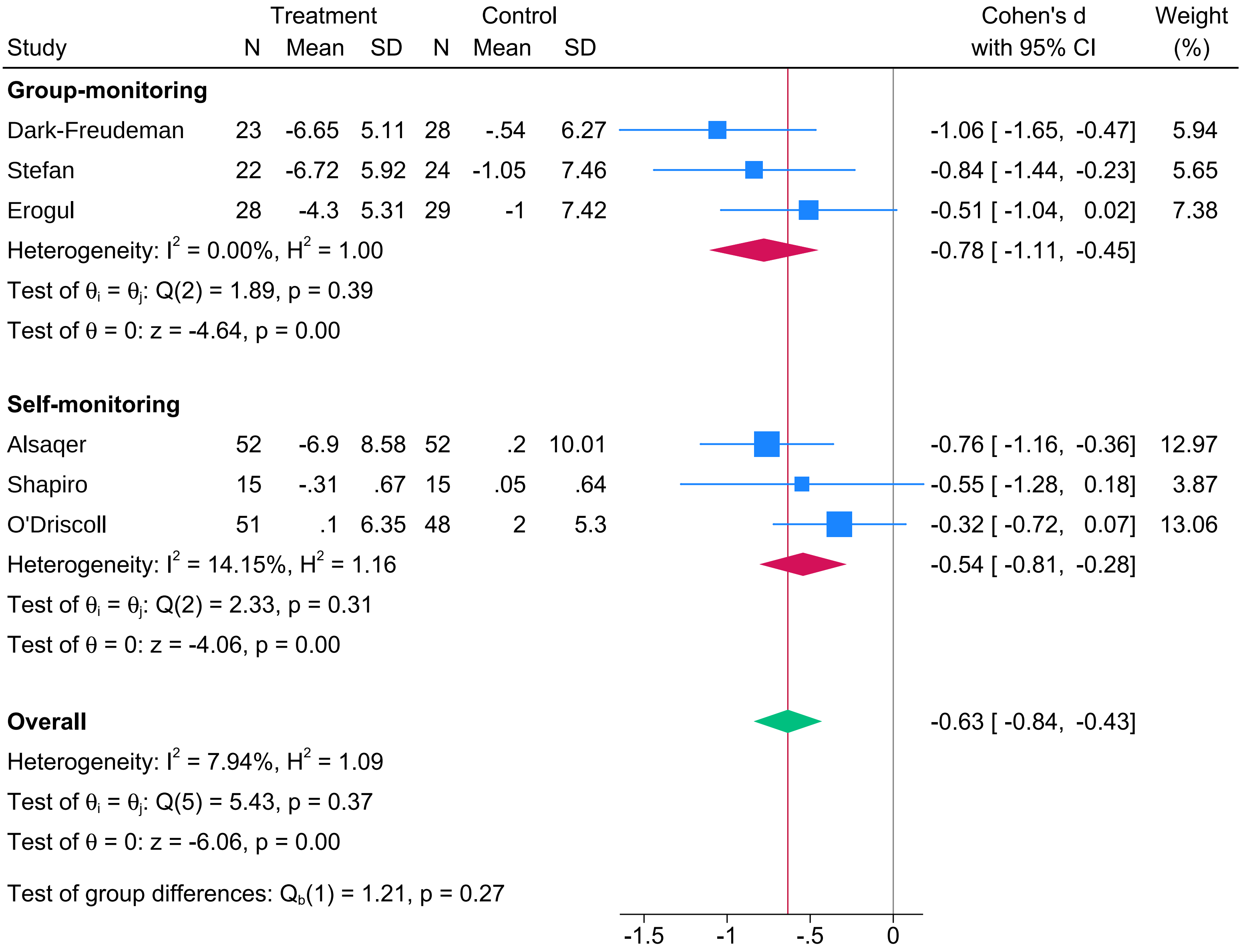

The Effect of Traditional Mindfulness on Stress in Adherence mechanism Subgroup
