# Supplementary material for: Online and traditional mindfulness-based interventions for stress in university students: a systematic review and meta-analysis versus control conditions
Source: Front Psychol. 2026 Mar 27;17:1755245. doi: 10.3389/fpsyg.2026.1755245 (PMC13067905; doi:10.3389/fpsyg.2026.1755245)
Supplement: Supplementary file 1 [file Data_Sheet_1.zip › Appendix 10.pdf]

Study	Treatment N	Treatment Mean	Treatment SD	Control N	Control Mean	Control SD	Cohen's d with 95% CI	Weight (%)
**≤4 weeks**								
Burger	32	8.5	17.23	28	-.3	17.45	0.51 [-0.01, 1.02]	22.21
Messer	23	12.78	19.6	49	2.85	15.27	0.59 [0.09, 1.10]	23.15
Heterogeneity: $I^2 = 0.00\%$, $H^2 = 1.00$							0.55 [0.19, 0.91]	
Test of $\theta_i = \theta_j$: Q(1) = 0.05, p = 0.82								
Test of $\theta = 0$: z = 3.00, p = 0.00								
**≥8 weeks**								
Alzahrani	39	.44	.97	45	.05	1.05	0.38 [-0.05, 0.82]	31.50
Hazlett-Stevens	25	7.1	14.48	43	-3.2	16.71	0.65 [0.14, 1.15]	23.15
Heterogeneity: $I^2 = 0.00\%$, $H^2 = 1.00$							0.50 [0.17, 0.82]	
Test of $\theta_i = \theta_j$: Q(1) = 0.59, p = 0.44								
Test of $\theta = 0$: z = 2.96, p = 0.00								
**Overall**							0.52 [0.28, 0.76]	
Heterogeneity: $I^2 = 0.00\%$, $H^2 = 1.00$								
Test of $\theta_i = \theta_j$: Q(3) = 0.70, p = 0.87								
Test of $\theta = 0$: z = 4.20, p = 0.00								
Test of group differences: $Q_b(1) = 0.05$, p = 0.82								

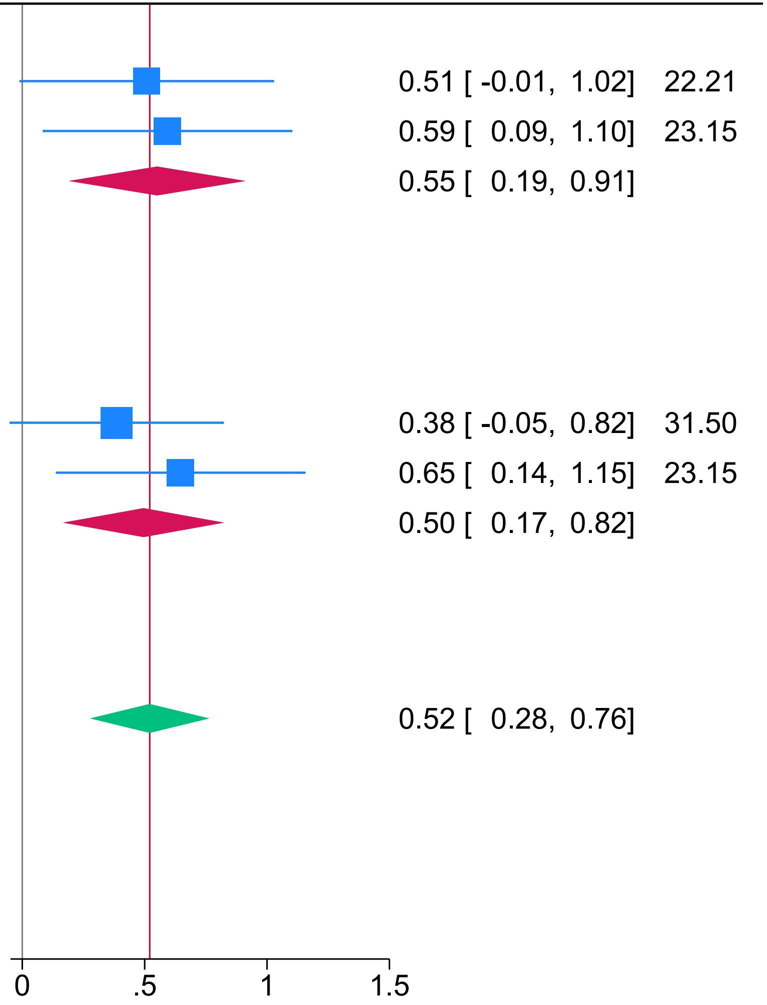

The Effect of Online Mindfulness on Mindfulness in Duration Subgroup

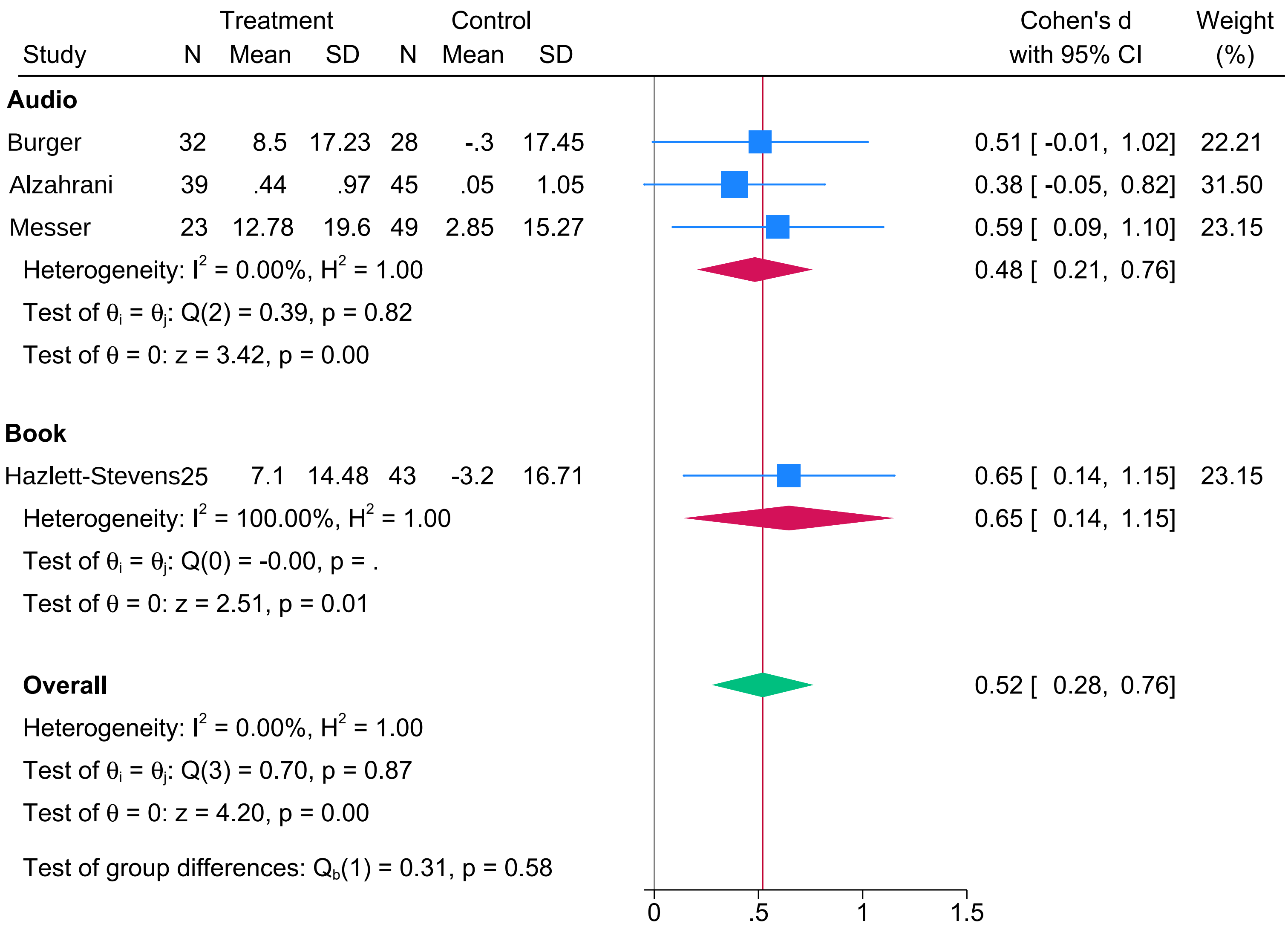

The Effect of Online Mindfulness on Mindfulness in Technology platform Subgroup

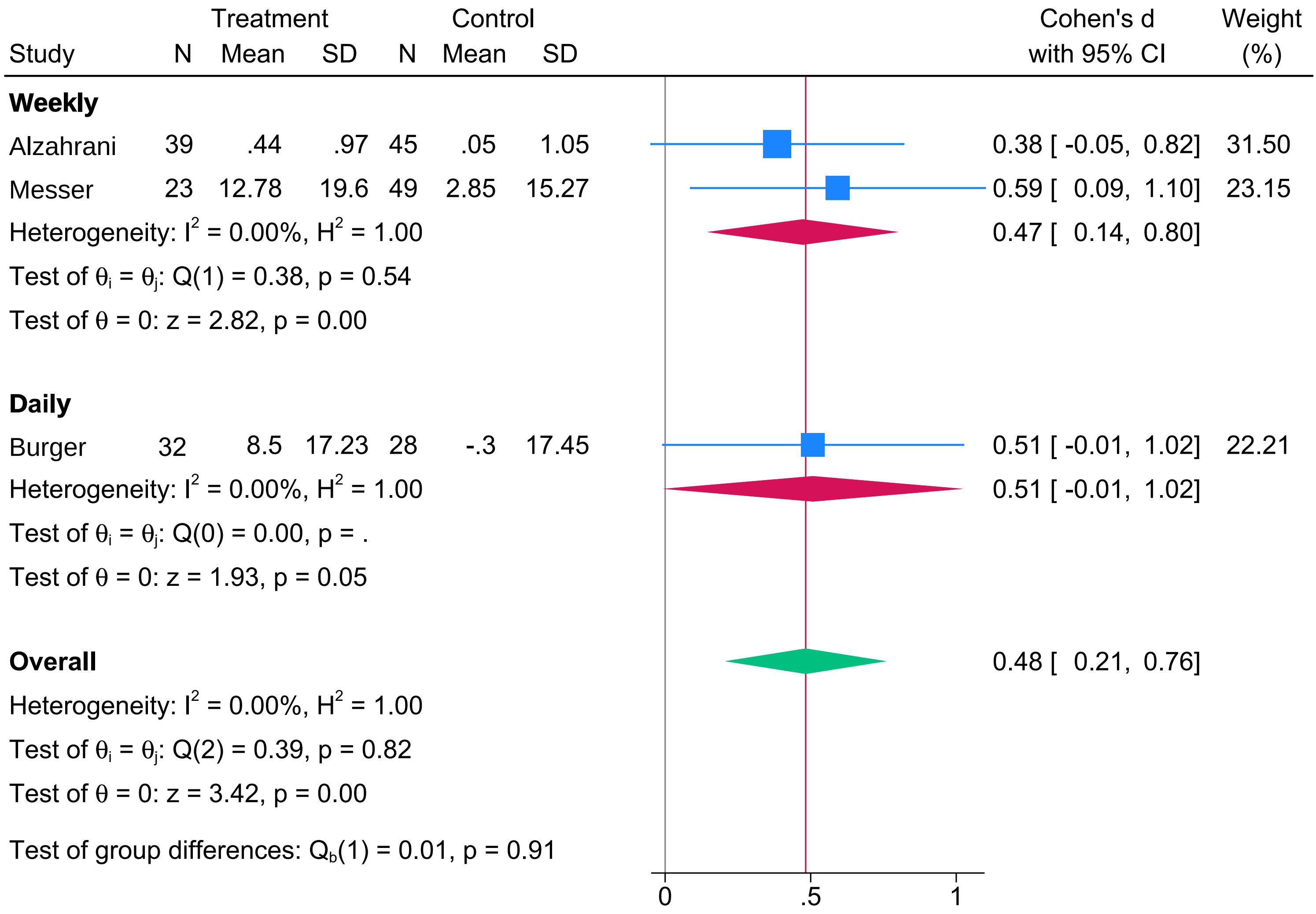

The Effect of Online Mindfulness on Mindfulness in Frequency Subgroup

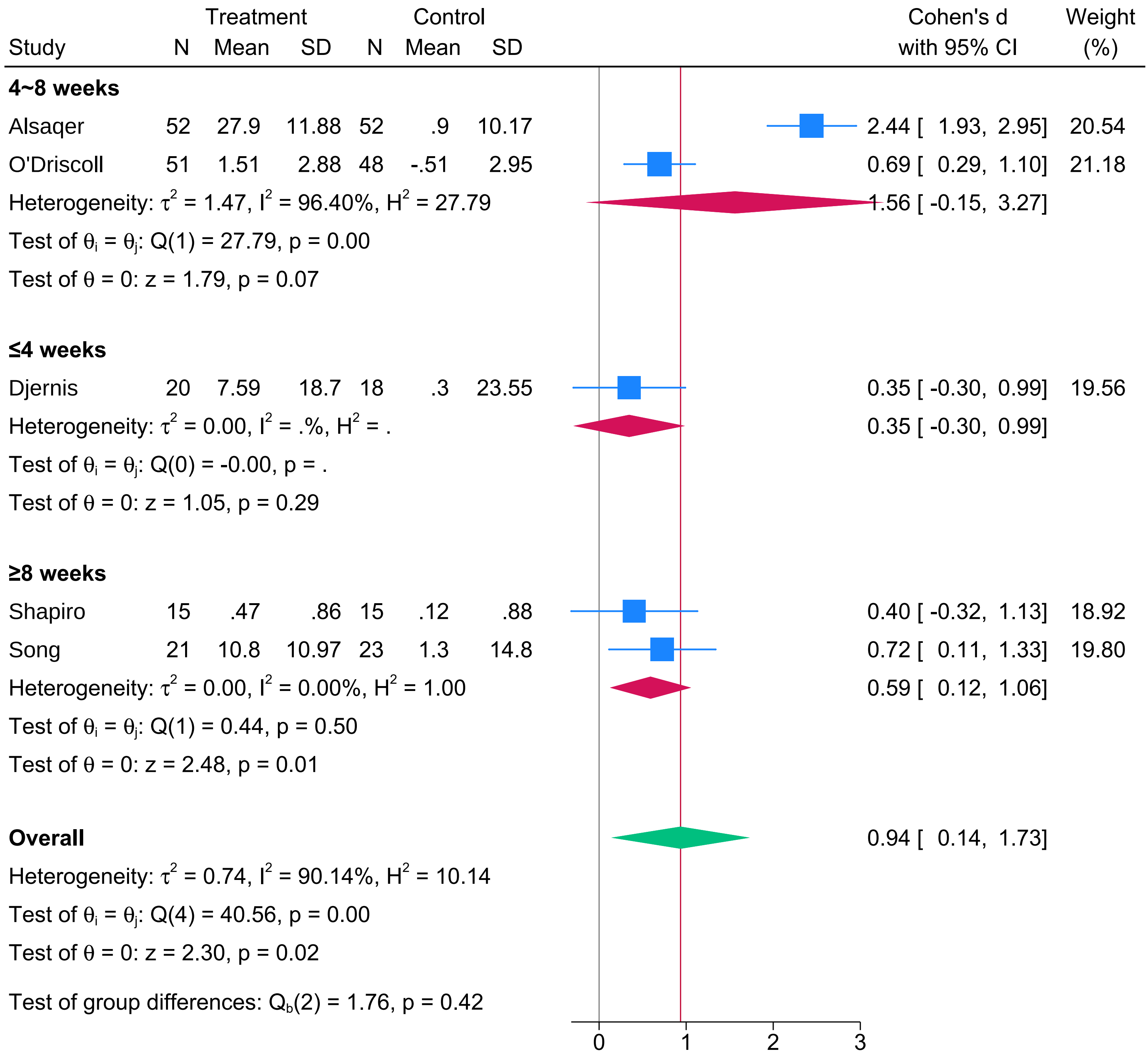

The Effect of Traditional Mindfulness on Mindfulness in Duration Subgroup
