# Supplementary material for: Online and traditional mindfulness-based interventions for stress in university students: a systematic review and meta-analysis versus control conditions
Source: Front Psychol. 2026 Mar 27;17:1755245. doi: 10.3389/fpsyg.2026.1755245 (PMC13067905; doi:10.3389/fpsyg.2026.1755245)
Supplement: Supplementary file 1 [file Data_Sheet_1.zip › Appendix 1.pdf]

Term index and search instructions

#	Search term[a]	Search target	Reference/function
1	Mindulness	Title and abstract and keyword	Eberth & Sedlmeier, 2012; Burton et al., 2017
2	Mindfulness-Based Stress Reduction	Title and abstract	Eberth & Sedlmeier, 2012; Burton et al., 2017
3	MBSR	Title and abstract	Eberth & Sedlmeier, 2012;Burton et al., 2017
4	#1 OR #2 OR #3		#1-3 compiled as intervention term
5	computer	Title and abstract	Smoktunowicz et al., 2020
6	tele	Title and abstract	Smoktunowicz et al., 2020
7	cyber	Title and abstract	Smoktunowicz et al., 2020
8	digital	Title and abstract	Smoktunowicz et al., 2020
9	online	Title and abstract	Smoktunowicz et al., 2020
10	web	Title and abstract	Smoktunowicz et al., 2020
11	smartphone	Title and abstract	Linardon, 2020
12	mobile	Title and abstract	Linardon, 2020
13	phone	Title and abstract	Linardon, 2020
14	application	Title and abstract	Gál et al., 2021
15	app	Title and abstract	Linardon, 2020； Gál et al., 2021
16	internet	Title and abstract	Smoktunowicz et al., 2020
17	virtual	Title and abstract	Smoktunowicz et al., 2020
18	VR	Title and abstract	Smoktunowicz et al., 2020

19	remote	Title and abstract	Smoktunowicz et al., 2020
20	distance	Title and abstract	Smoktunowicz et al., 2020
21	videoconference	Title and abstract	Smoktunowicz et al., 2020
22	e-mail	Title and abstract	Smoktunowicz et al., 2020
23	e-health	Title and abstract	Smoktunowicz et al., 2020
24	guided self-help	Title and abstract	Smoktunowicz et al.,2020
25	self-help through the internet	Title and abstract	Smoktunowicz et al., 2020
26	#5 OR #6 OR #6 OR #7 OR #8 OR #9 OR #10 OR #11 OR #12 OR #13 OR #14 OR #15 OR #16 OR #17 OR #18 OR #19 OR #20 OR #21 OR #22 OR #23 OR #24 OR #25		#5-25 compiled as delivery modality term
27	stress	Title and abstract	Burton et al., 2017
28	#27		#27 as targeted interventions term
29	student	Title and abstract	Increase specificity by defining intervention populations of interest
30	#29		#29 as included samples
31	primary school student	Title and abstract	Exclusion of non-eligible groups increases the precision of intervention targeting.
32	elementary school student	Title and abstract	Exclusion of non-eligible groups increases the precision of intervention targeting.

33	Secondary school student	Title and abstract	Exclusion of non-eligible groups increases the precision of intervention targeting.
34	middle school student	Title and abstract	Exclusion of non-eligible groups increases the precision of intervention targeting.
35	junior school student	Title and abstract	Exclusion of non-eligible groups increases the precision of intervention targeting.
36	junior high school student	Title and abstract	Exclusion of non-eligible groups increases the precision of intervention targeting.
37	junior middle school student	Title and abstract	Exclusion of non-eligible groups increases the precision of intervention targeting.
38	#31 OR #32 OR #33 OR #34 OR #35 OR #36 OR #37		#31-37 compiled as excluded samples
39	#4 AND #28 AND #30 NOT #26 NOT #38		The terms of traditional mindfulness have been compiled.
40	#4 AND #26 AND #28 AND #30 NOT #38		The terms of digital mindfulness have been compiled.

Embase

Online minfulness

#1.('mindfulness'/exp OR 'mindfulness-based stress reduction'/exp OR mbsr OR 'mindfulness':ti,ab,kw OR 'mindfulness-based stress reduction':ti,ab,kw)

#2. ('computer'/exp OR 'telemedicine'/exp OR 'digital health'/exp OR 'online system'/exp OR 'mobile application'/exp OR 'internet'/exp OR 'virtual reality'/exp OR computer*:ti,ab,kw OR tele*:ti,ab,kw OR cyber*:ti,ab,kw OR digital*:ti,ab,kw OR online*:ti,ab,kw OR web:ti,ab,kw OR smartphone*:ti,ab,kw OR mobile:ti,ab,kw OR phone:ti,ab,kw OR application:ti,ab,kw OR app:ti,ab,kw OR internet*:ti,ab,kw OR virtual*:ti,ab,kw OR vr:ti,ab,kw OR remote*:ti,ab,kw OR distance*:ti,ab,kw OR

videoconference*:ti,ab,kw OR 'e-mail':ti,ab,kw OR 'e-health':ti,ab,kw OR 'guided self-help':ti,ab,kw OR 'self-help through the internet':ti,ab,kw)
#3. ('stress'/exp OR stress:ti,ab,kw)
#4. ('student'/exp OR student*:ti,ab,kw)
#5. NOT ('primary school student':ti OR 'elementary school student':ti OR 'secondary school student':ti OR 'middle school student':ti OR 'junior school student':ti OR 'junior high school student':ti OR 'junior middle school student':ti
#6. [2010-01-01:2025-05-01]/dp
#7. #1 AND #2 AND #3 AND #4 AND #5 AND #6

Traditionnal minfulness
#1.('mindfulness'/exp OR mindfulness*:ti,ab,kw OR 'mindfulness-based stress reduction':ti,ab,kw OR mbsr:ti,ab,kw)
#2. ('stress'/exp OR stress:ti,ab,kw)
#3. ('student'/exp OR student*:ti,ab,kw)
#4. NOT(computer* OR tele* OR cyber* OR digital* OR online* OR web OR smartphone* OR mobile OR phone OR application OR app OR internet* OR virtual* OR vr OR remote* OR distance* OR videoconference* OR 'e-mail' OR 'e-health' OR 'guided self-help' OR 'self-help through the internet'):ti,ab,kw
#5. NOT('primary school student' OR 'elementary school student' OR 'secondary school student' OR 'middle school student' OR 'junior school student' OR 'junior high school student' OR 'junior middle school student')
#6. #1 AND #2 AND #3 AND #4 AND #5

Wos
Online minfulness
#1. TS=(Mindfulness OR "Mindfulness-Based Stress Reduction" OR MBSR)
#2. TS=(computer* OR tele* OR cyber* OR digital* OR online* OR web OR smartphone* OR mobile OR phone OR application OR app OR internet* OR virtual* OR VR OR remote* OR distance* OR videoconference* OR e-mail OR "e-health" OR "guided self-help" OR "self-help through the internet")
#3. TS=(stress)
#4. TS=(student*)
#5. NOT(TI=("primary school student") OR TI=("elementary school student") OR TI=("Secondary school student") OR TI=("middle school student") OR TI=("junior school student") OR TI=("Junior high school student") OR TI=("junior middle school student"))
#6. #1 AND #2 AND #3 AND #4 AND #5

Traditionnal minfulness
#1. TS=(Mindfulness* OR Mindfulness-Based Stress Reduction OR MBSR*)
#2. TS=(stress)
#3. TS=(student*)
#4. NOT(TI=("primary school student") OR TI=("elementary school student") OR TI=("Secondary school student") OR TI=("middle school student") OR TI=("junior school student") OR TI=("Junior high school student") OR TI=("junior middle school student"))
#5. NOT(TS=(computer* OR tele* OR cyber* OR digital* OR online* OR web OR smartphone* OR mobile OR phone OR application OR app OR internet* OR virtual* OR VR OR remote* OR distance* OR videoconference* OR e-mail OR "e-health" OR "guided self-help" OR "self-help through the internet"))
#6. #1 AND #2 AND #3 AND #4 AND #5

Psynifo
Online minfulness
#1. (exp Mindfulness/ OR "Mindfulness-Based Stress Reduction".mp. OR MBSR.mp. OR Mindfulness.ti,ab,id OR "Mindfulness-Based Stress Reduction".ti,ab,id OR MBSR.ti,ab,id)
#2. (exp Stress/ OR stress.ti,ab,id)
#3. (exp Students/ OR student.ti,ab,id)
#4. (computer OR tele* OR cyber* OR digital* OR online* OR web OR smartphone* ORmobile OR phone OR application OR app OR internet* OR virtual* OR VR OR remote* OR distance* OR videoconferenc* OR e-mail OR "e-health" OR "guided self-help" OR "self-help through the internet").mp.)
#5. NOT("primary school student".ti O"elementary school student".ti O"Secondary school student".ti OR "middle school student".ti OR "junior school student".ti OR "Junior high school student".ti OR "junior middle school student".ti)
#6. #1 AND #2 AND #3 AND #4 AND #5

Traditional minfulness
#1. (exp Mindfulness/ OR "Mindfulness-Based Stress Reduction".mp. OR MBSR.mp. OR Mindfulness.ti,ab,id OR "Mindfulness-Based Stress Reduction".ti,ab,id OR MBSR.ti,ab,id)
#2. (exp Stress/ OR stress.ti,ab,id)
#3. (exp Students/ OR student.ti,ab,id)
#4. NOT(computer OR tele* OR cyber* OR digital* OR online* OR web OR smartphone* ORmobile OR phone OR application OR app OR internet* OR virtual*

OR VR OR remote* OR distance* OR videoconferenc* OR e-mail OR "e-health" OR "guided self-help" OR "self-help through the internet").mp.)
#5. NOT("primary school student".ti O"elementary school student".ti O"Secondary school student".ti OR "middle school student".ti OR "junior school student".ti OR "Junior high school student".ti OR "junior middle school student".ti)
#6. #1 AND #2 AND #3 AND #4 AND #5

Medline

Online minfulness
#1. (exp Mindfulness/ OR "Mindfulness-Based Stress Reduction".mp. OR MBSR.mp. OR Mindfulness.ti,ab. OR "Mindfulness-Based Stress Reduction".ti,ab. OR MBSR.ti,ab.)
#2. (exp Stress, Psychological/ OR stress.mp.)
#3. (exp Students/ OR student*.mp.)
#4. (computer* OR tele* OR cyber* OR digital* OR online* OR web OR smartphone* OR mobile OR phone OR application OR app OR internet* OR virtual* OR VR OR remote* OR distance* OR videoconferenc* OR e-mail OR "e-health" OR "guided self-help" OR "self-help through the internet".mp.)
#5. NOT("primary school student".ti OR "elementary school student".ti OR "Secondary school student".ti OR "middle school student".ti OR "junior school student".ti OR "Junior high school student".ti OR "junior middle school student".ti OR "high school".ti OR "elementary school".ti OR "secondary school".ti)
#6. #1 AND #2 AND #3 AND #4 AND #5

Traditional minfulness
#1. (exp Mindfulness/ OR "Mindfulness-Based Stress Reduction".mp. OR MBSR.mp. OR Mindfulness.ti,ab. OR "Mindfulness-Based Stress Reduction".ti,ab. OR MBSR.ti,ab.)
#2. (exp Stress, Psychological/ OR stress.mp.)
#3. (exp Students/ OR student*.mp.)
#4. NOT(computer* OR tele* OR cyber* OR digital* OR online* OR web OR smartphone* OR mobile OR phone OR application OR app OR internet* OR virtual* OR VR OR remote* OR distance* OR videoconferenc* OR e-mail OR "e-health" OR "guided self-help" OR "self-help through the internet".mp.)
#5. NOT("primary school student".ti OR "elementary school student".ti OR "Secondary school student".ti OR "middle school student".ti OR "junior school student".ti OR "Junior high school student".ti OR "junior middle school student".ti OR "high school".ti OR "elementary school".ti OR "secondary school".ti)
#6. #1 AND #2 AND #3 AND #4 AND #5

References

Eberth, J., & Sedlmeier, P. (2012). The effects of mindfulness meditation: a meta-analysis. Mindfulness, 3(3), 174-189.https://doi.org/10.1007/s12671-012-0101-x

Burton, A., Burgess, C., Dean, S., Koutsopoulou, G. Z., & Hugh-Jones, S. (2017). How effective are mindfulness-based interventions for reducing stress among healthcare professionals? A systematic review and meta-analysis. Stress and Health, 33(1), 3-13. https://doi.org/10.1002/smi.2673

Smoktunowicz, E., Barak, A., Andersson, G., Banos, R. M., Berger, T., Botella, C., ... & Carlbring, P. (2020). Consensus statement on the problem of terminology in psychological interventions using the internet or digital components. Internet Interventions, 21, 100331. https://doi.org/10.1016/j.invent.2020.100331

Linardon, J. (2020). Can acceptance, mindfulness, and self-compassion be learned by smartphone apps? A systematic and meta-analytic review of randomized controlled trials. Behavior Therapy, 51(4), 646-658. https://doi.org/10.1016/j.beth.2019.10.002

Gál, É., Ștefan, S., & Cristea, I. A. (2021). The efficacy of mindfulness meditation apps in enhancing users' well-being and mental health related outcomes: a meta-analysis of randomized controlled trials. Journal of affective disorders, 279, 131-142. https://doi.org/10.1016/j.jad.2020.09.134
