# Supplementary figures and images for: Online and traditional mindfulness-based interventions for stress in university students: a systematic review and meta-analysis versus control conditions
Source: Front Psychol. 2026 Mar 27;17:1755245. doi: 10.3389/fpsyg.2026.1755245 (PMC13067905; doi:10.3389/fpsyg.2026.1755245)

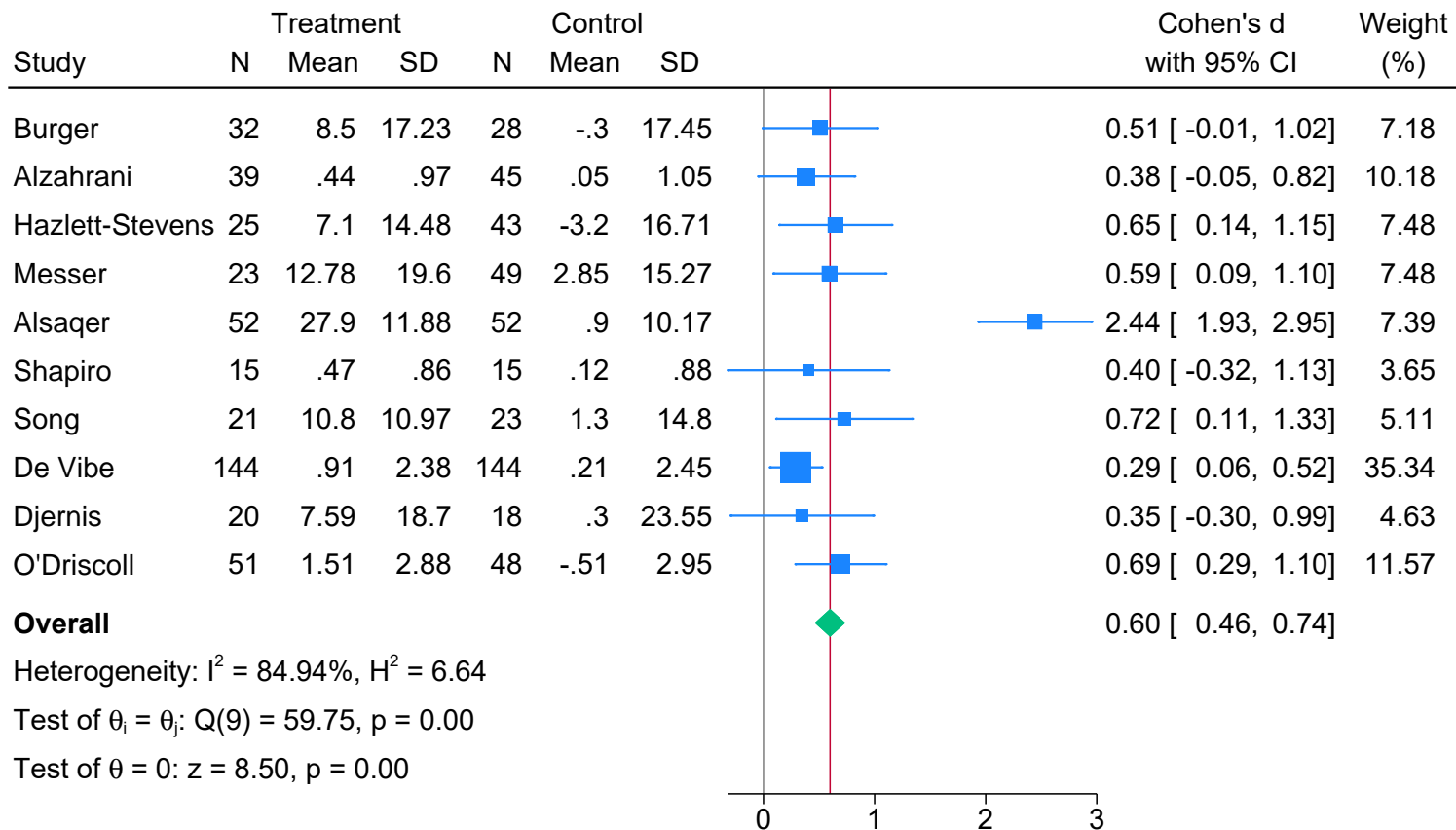

Supplement: Supplementary file 1 [file Data_Sheet_1.zip › Appendix 7.pdf]

## Galbraith plot (k=10)

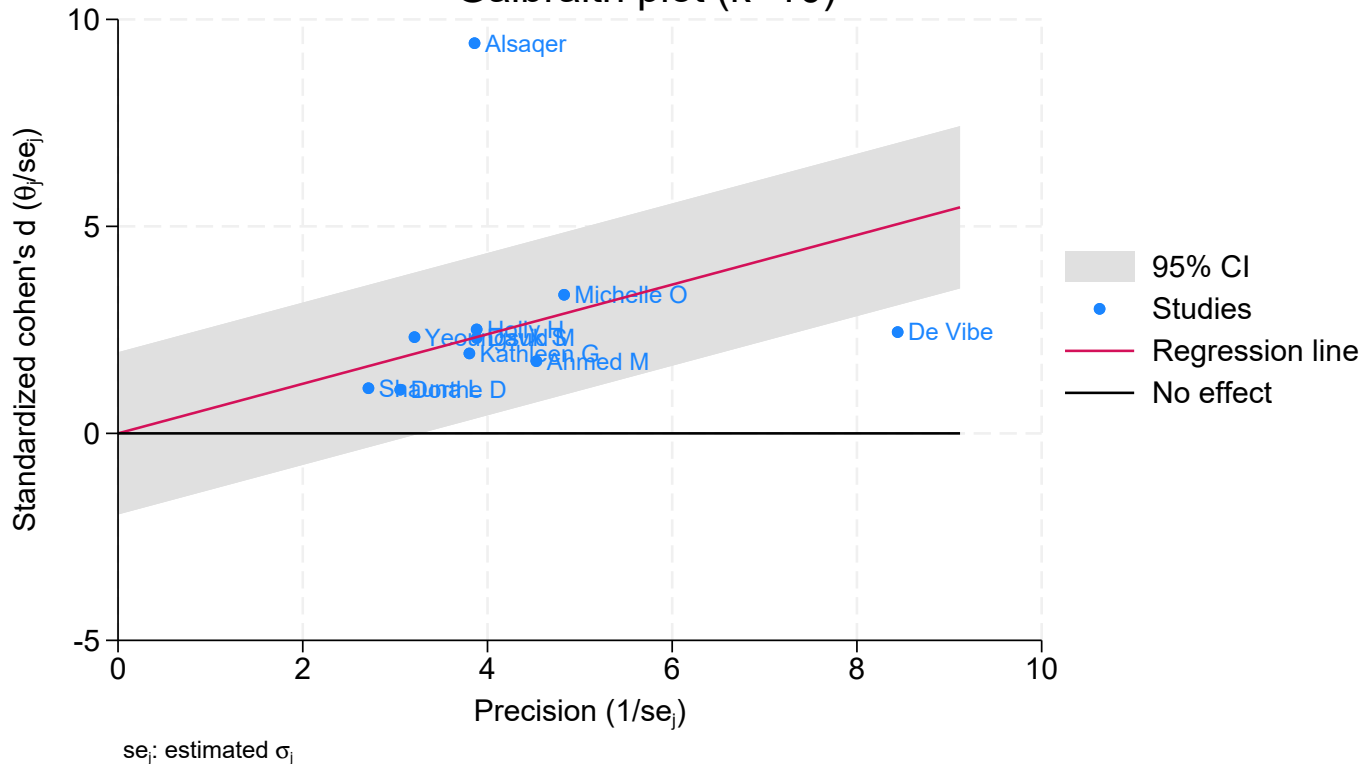

Supplement: Supplementary file 1 [file Data_Sheet_1.zip › Appendix 8.pdf]

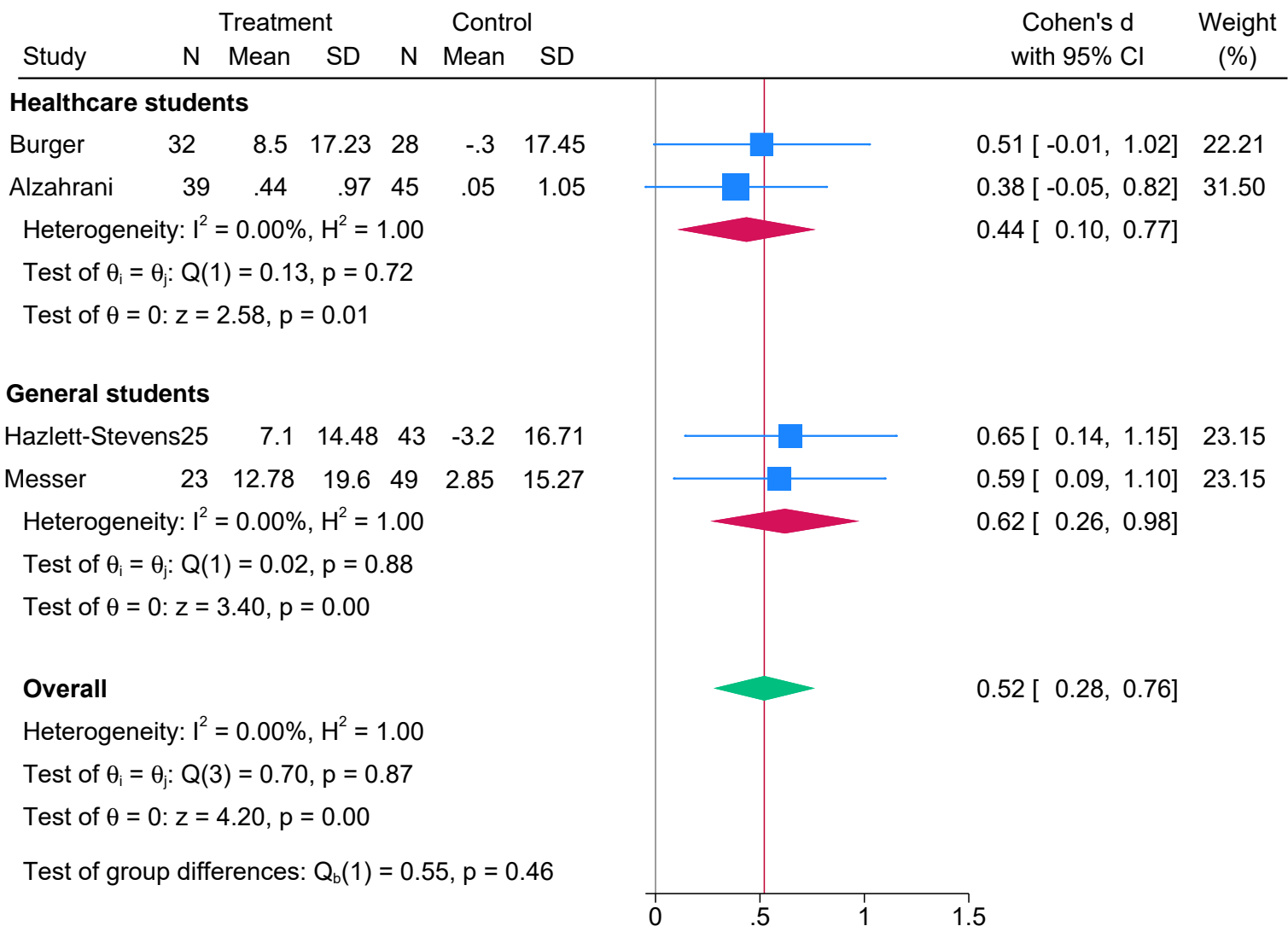

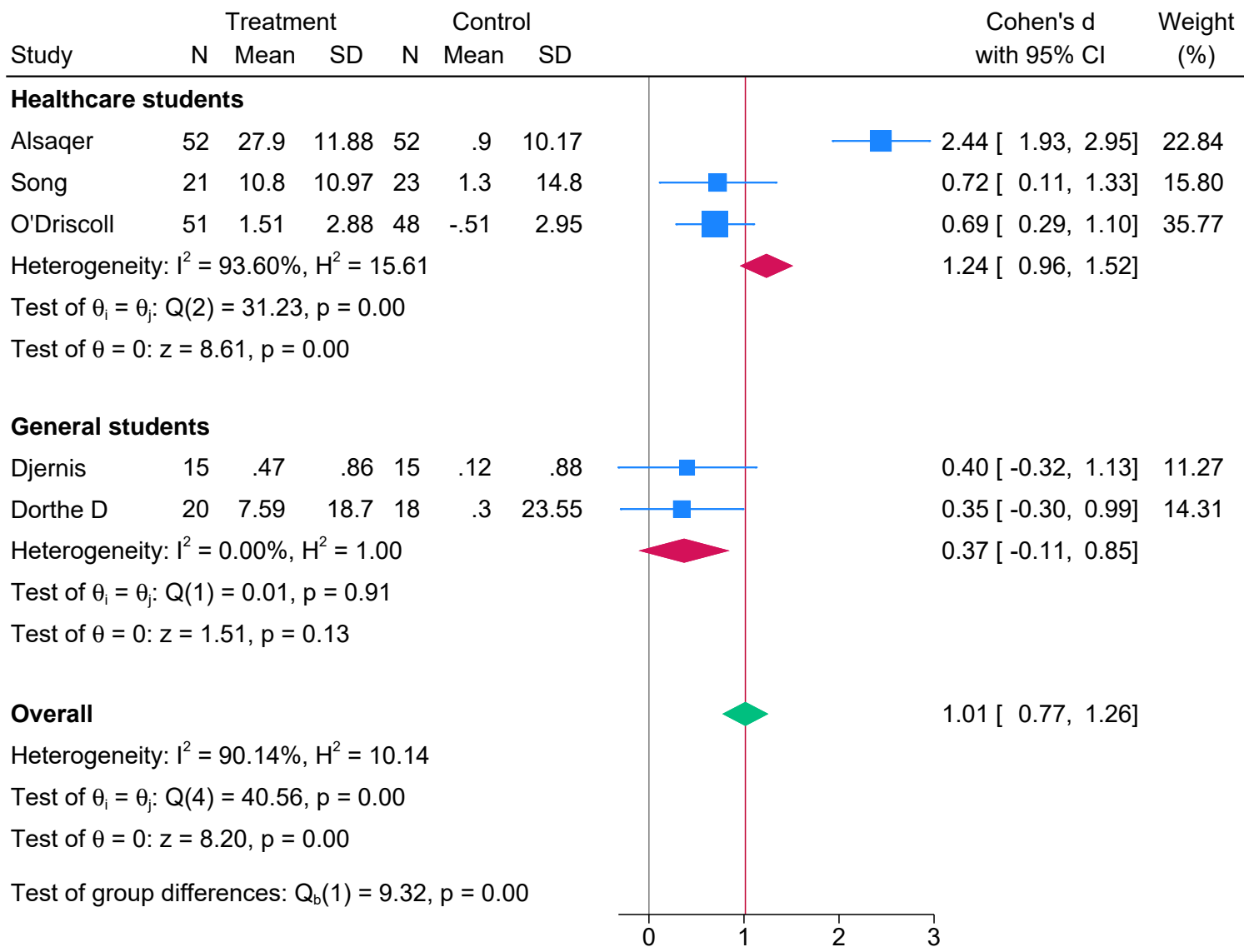

The Effect of Traditional Mindfulness on Mindfulness in Student Subgroup

Supplement: Supplementary file 1 [file Data_Sheet_1.zip › Appendix 9.pdf]

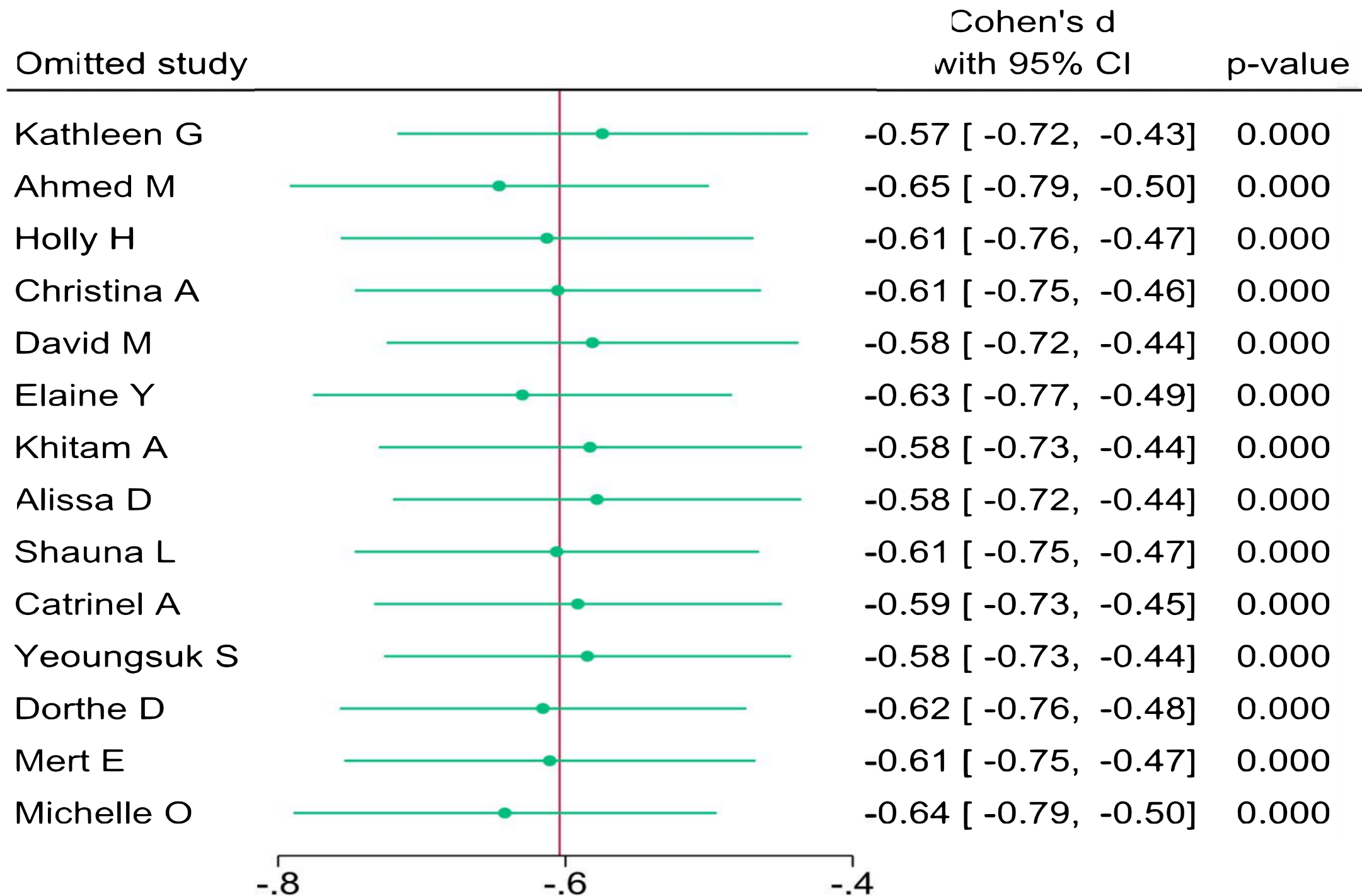

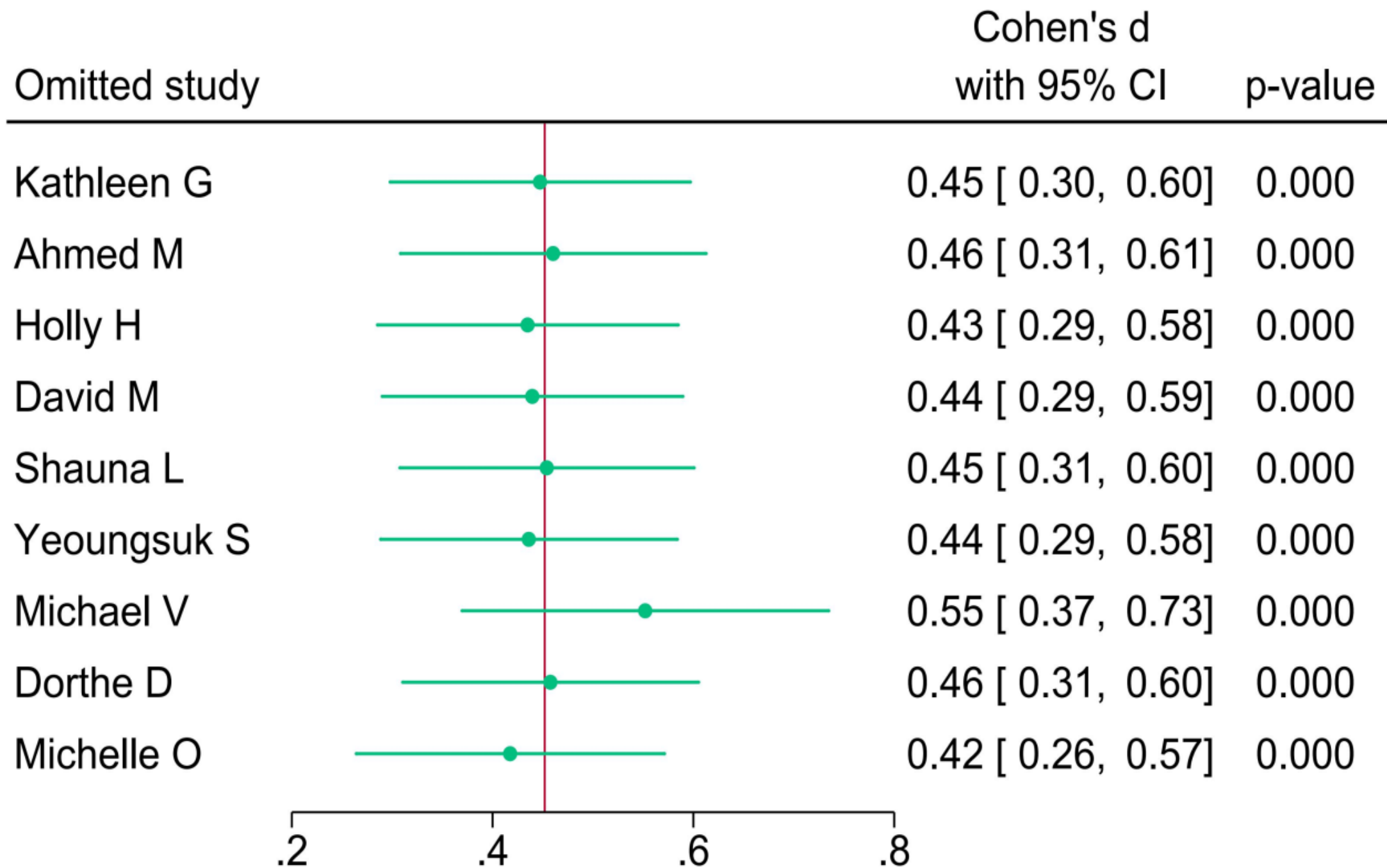

Supplement: Supplementary file 1 [file Data_Sheet_1.zip › Appendix 11.pdf]

## Funnel plot

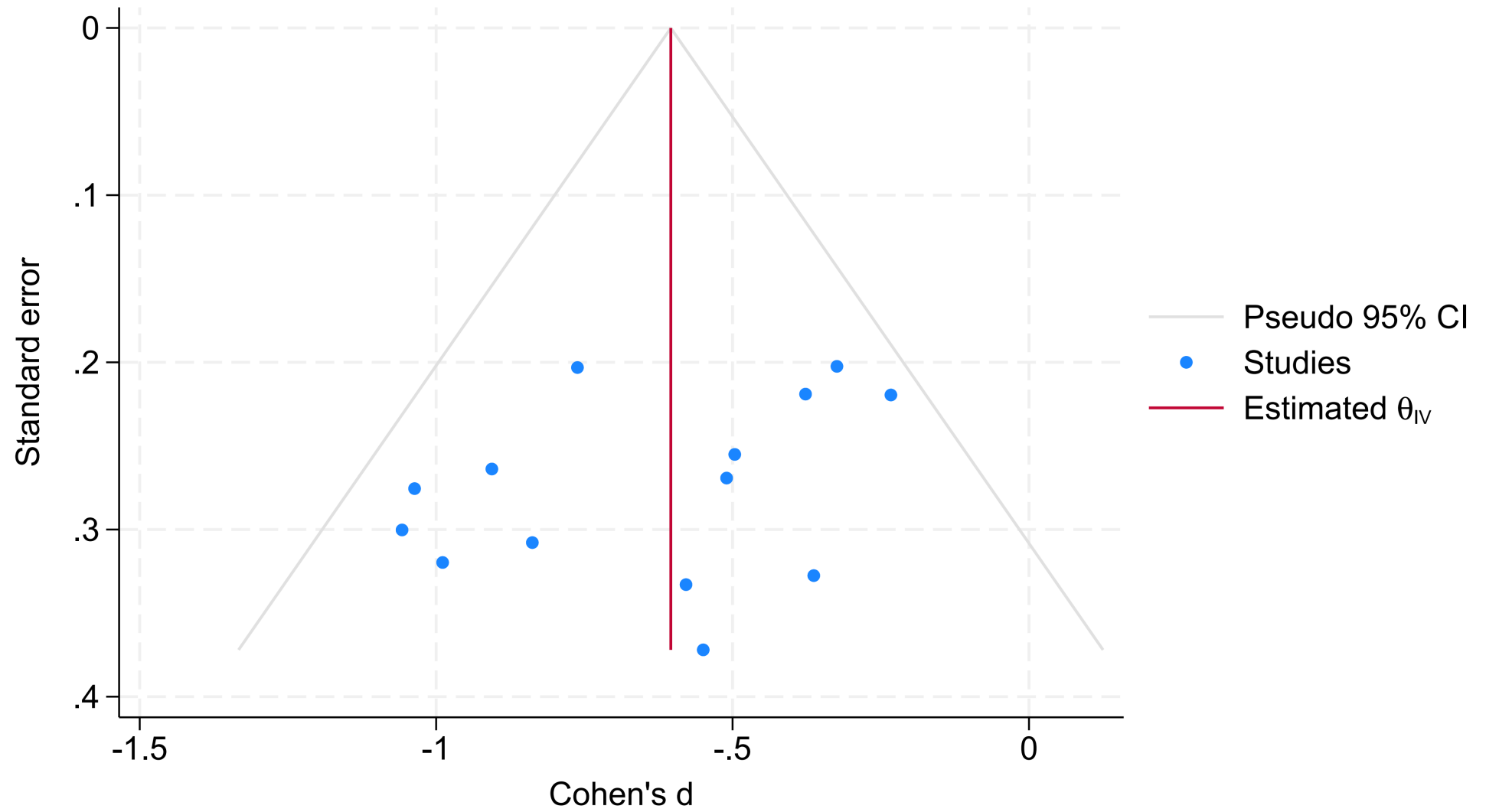

## Funnel plot

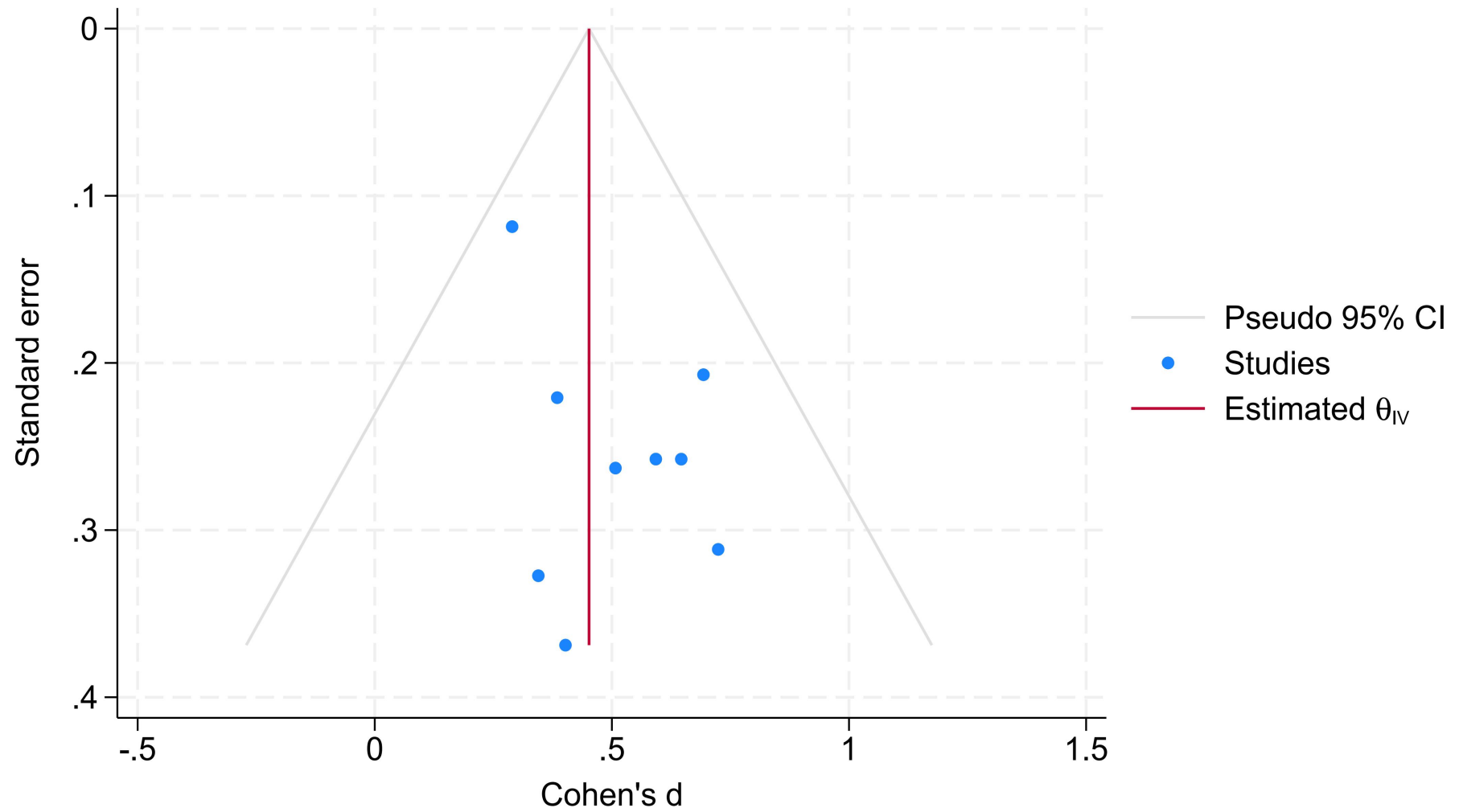

Supplement: Supplementary file 1 [file Data_Sheet_1.zip › Appendix 12.pdf]

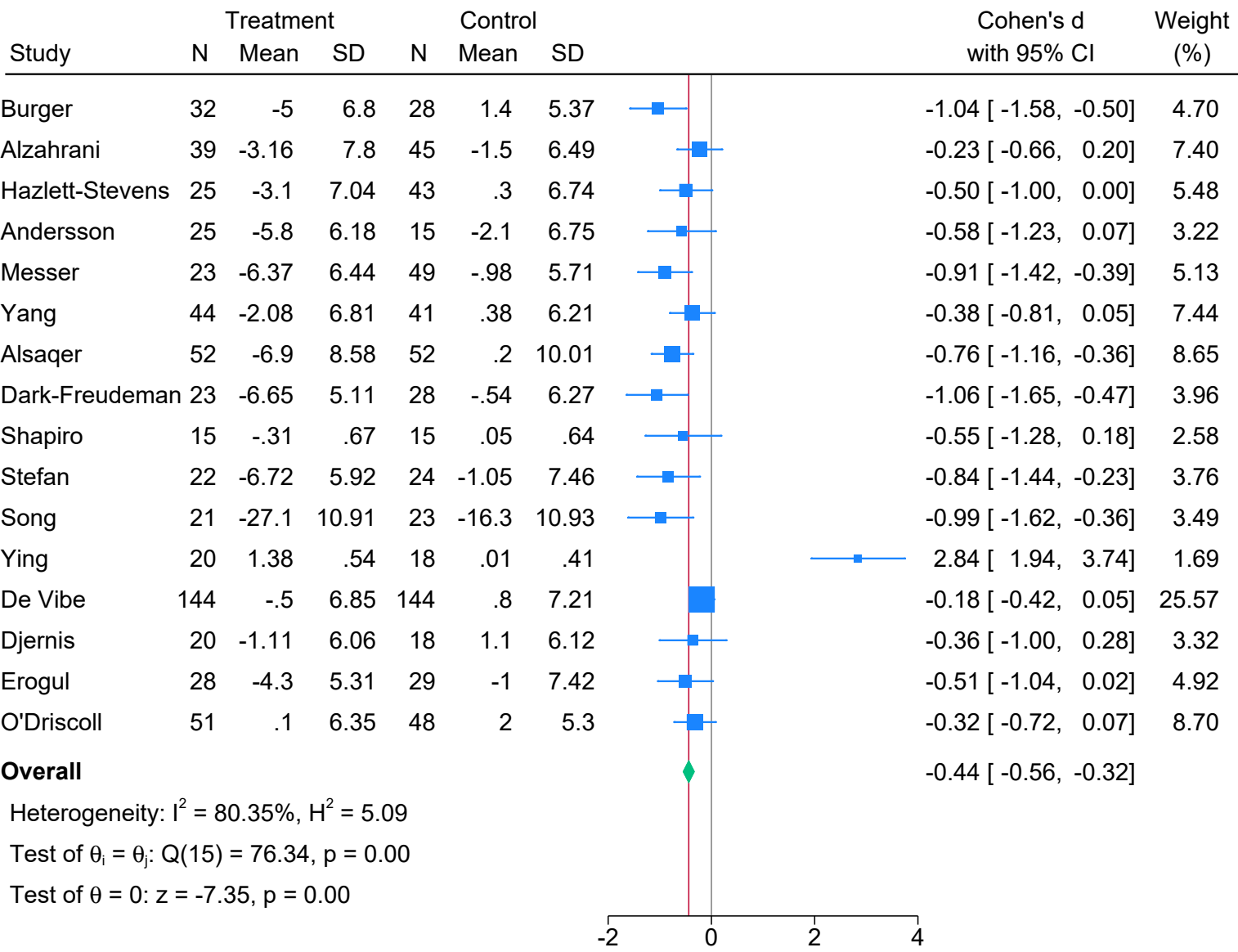

Appendix 1: Forest plot for stress (k=16)

Supplement: Supplementary file 1 [file Data_Sheet_1.zip › Appendix 2.pdf]

# Galbraith plot (k=16)

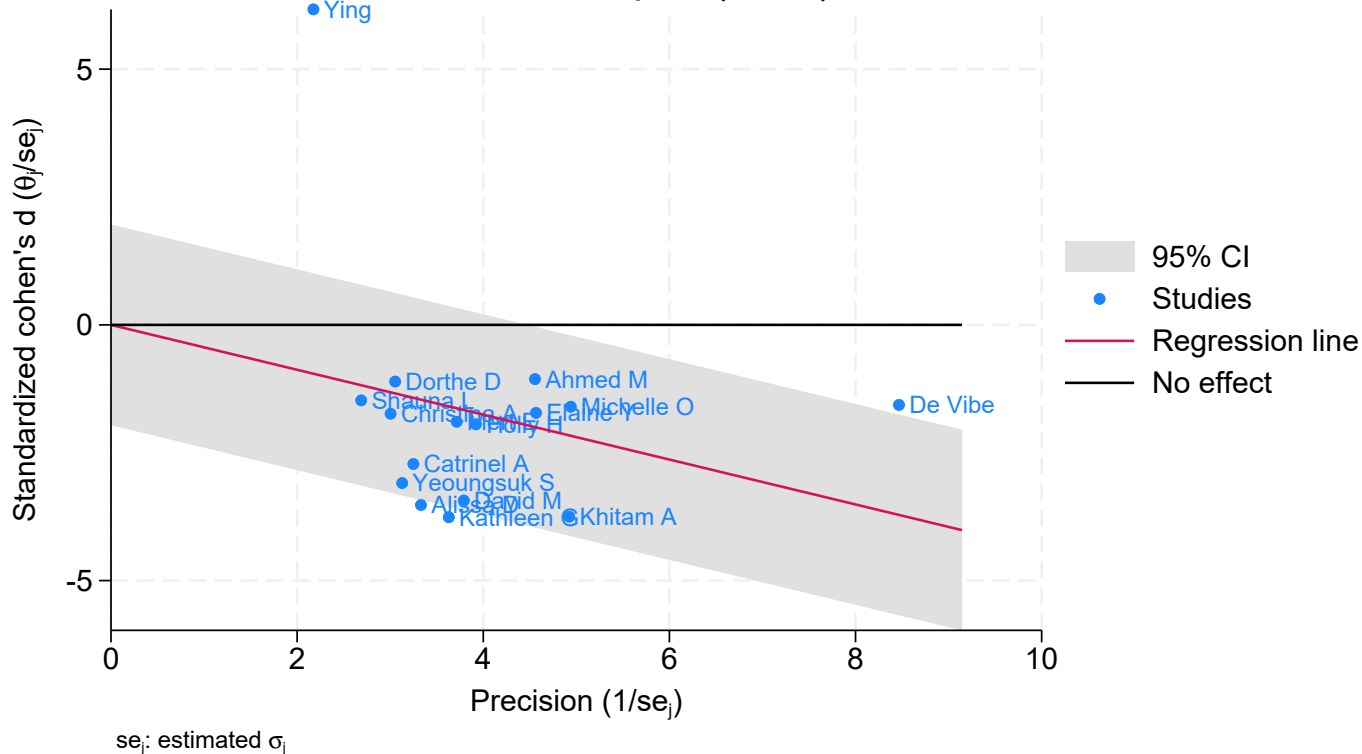

Supplement: Supplementary file 1 [file Data_Sheet_1.zip › Appendix 3.pdf]

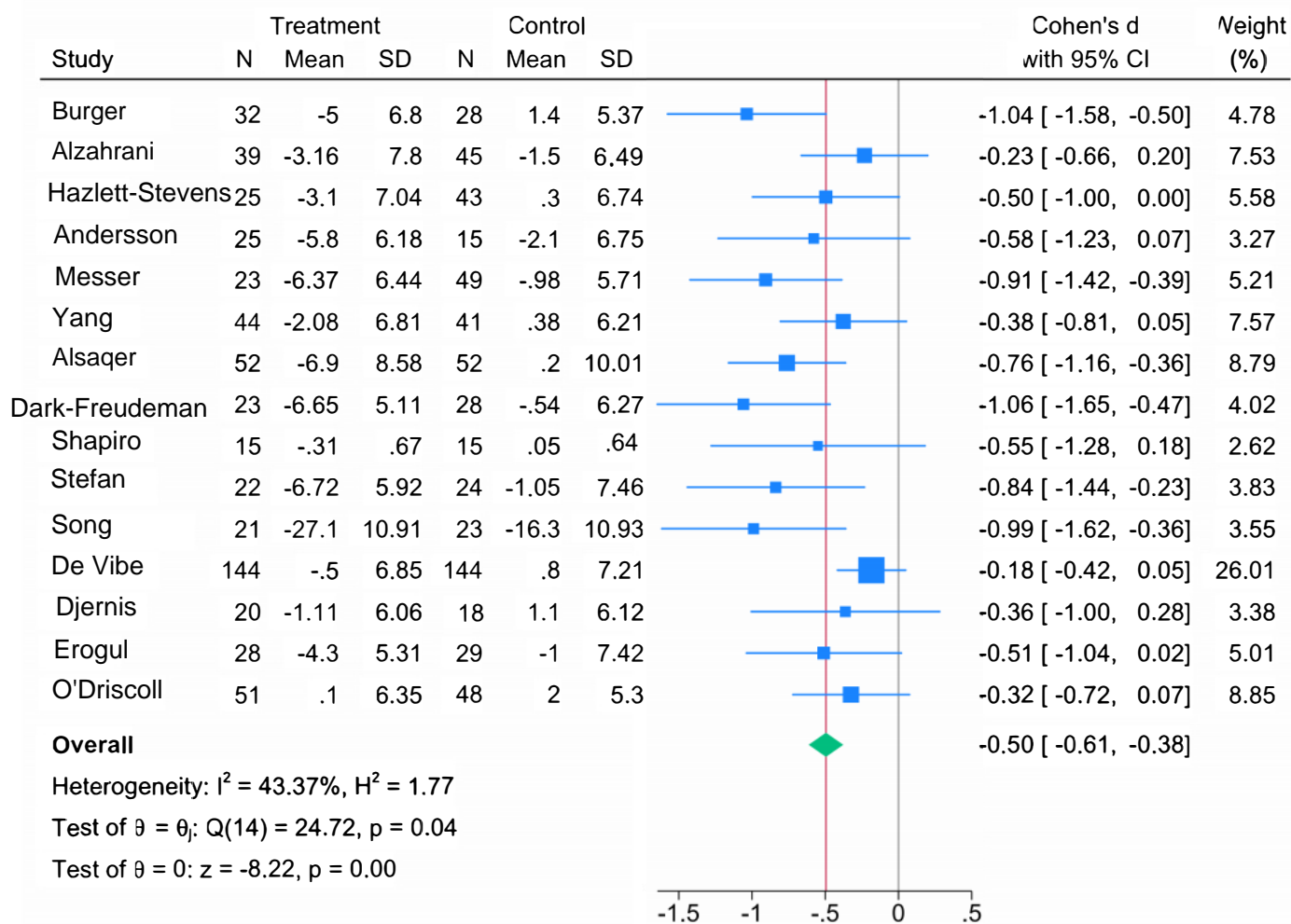

Fixed-effects inverse-variance model

Supplement: Supplementary file 1 [file Data_Sheet_1.zip › Appendix 4.pdf]

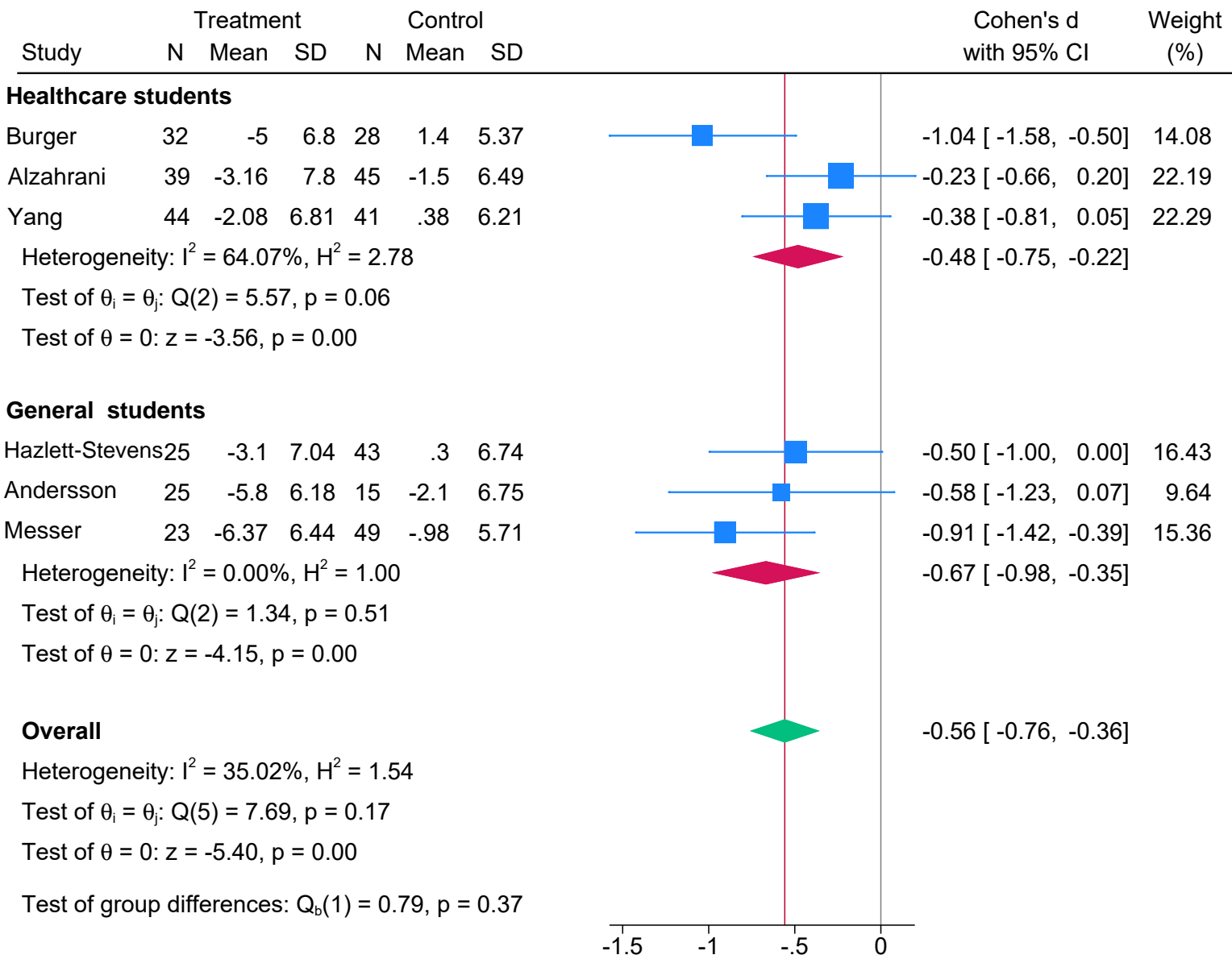

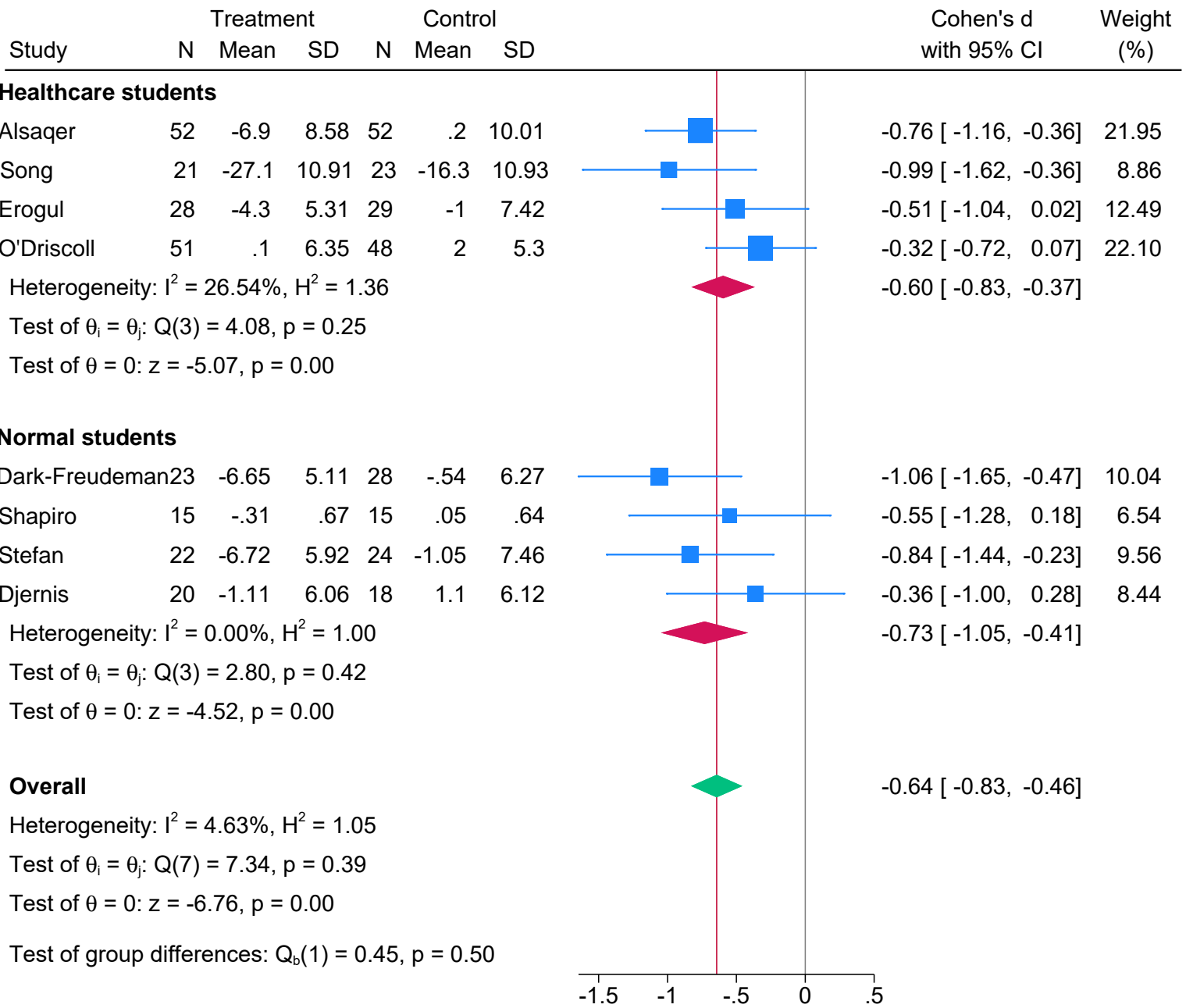

The Effect of Traditional Mindfulness on Stress in Student Subgroups

Supplement: Supplementary file 1 [file Data_Sheet_1.zip › Appendix 5.pdf]
